# Shedding light on neurons: optical approaches for neuromodulation

**DOI:** 10.1093/nsr/nwac007

**Published:** 2022-01-18

**Authors:** Shan Jiang, Xiang Wu, Nicholas J Rommelfanger, Zihao Ou, Guosong Hong

**Affiliations:** Department of Materials Science and Engineering, Stanford University, Stanford, CA 94305, USA; Wu Tsai Neurosciences Institute, Stanford University, Stanford, CA 94305, USA; Department of Materials Science and Engineering, Stanford University, Stanford, CA 94305, USA; Wu Tsai Neurosciences Institute, Stanford University, Stanford, CA 94305, USA; Wu Tsai Neurosciences Institute, Stanford University, Stanford, CA 94305, USA; Department of Applied Physics, Stanford University, Stanford, CA 94305, USA; Department of Materials Science and Engineering, Stanford University, Stanford, CA 94305, USA; Wu Tsai Neurosciences Institute, Stanford University, Stanford, CA 94305, USA; Department of Materials Science and Engineering, Stanford University, Stanford, CA 94305, USA; Wu Tsai Neurosciences Institute, Stanford University, Stanford, CA 94305, USA

**Keywords:** optical neuromodulation, optogenetics, neural interfaces

## Abstract

Today's optical neuromodulation techniques are rapidly evolving, benefiting from advances in photonics, genetics and materials science. In this review, we provide an up-to-date overview of the latest optical approaches for neuromodulation. We begin with the physical principles and constraints underlying the interaction between light and neural tissue. We then present advances in optical neurotechnologies in seven modules: conventional optical fibers, multifunctional fibers, optical waveguides, light-emitting diodes, upconversion nanoparticles, optical neuromodulation based on the secondary effects of light, and unconventional light sources facilitated by ultrasound and magnetic fields. We conclude our review with an outlook on new methods and mechanisms that afford optical neuromodulation with minimal invasiveness and footprint.

## Teaser

This review article provides a comprehensive overview of recent advances in optical neuromodulation in the context of addressing various physical constraints of in vivo light delivery.

## INTRODUCTION

Understanding neural computation requires causal manipulation of neural activity to dissect the complex circuit connections and the relationship between neural activity and certain behaviors. Arguably, the first demonstration of neuromodulation for deconstructing the causal relationship between structures and functions was realized by the Italian scientist Luigi Galvani in the late 18th century to stimulate the sciatic nerve of a frog. Modern electrical stimulation techniques offer many insights into the cognitive functions of specific circuits and structures in the nervous system. However, there remain two fundamental challenges of electrical stimulation: first, no technique has yet succeeded in spatially confining the electric field down to a single neuron; second, electrical stimulation lacks neuron-type specificity to manipulate a subgroup of neurons with a particular molecular signature. To this end, optical stimulation addresses these two challenges owing to the sophisticated physical approach of confining the light focus, and the molecular biology strategy of genetically modifying specific neurons with light-sensitive proteins.

Scientists have long sought to modulate neural activity with light. Besides naturally light-responsive cells such as photoreceptors and intrinsically photosensitive retinal ganglion cells (ipRGCs) in the retina, it has been known for more than a century that light influences the activity of neurons that are not specialized to be light sensitive [1]. The invention of lasers in the 1960s revitalized the research area of optical stimulation in nerve cells, with the first demonstration of laser stimulation of abdominal ganglion neurons in aplysia in the early 1970s [2]. The development of photochemistry has provided neuroscientists with another toolbox for optical neuromodulation. The light-triggered uncaging of neurotransmitters, such as glutamate, has been used to analyze neural networks since the 1990s [3]. However, all these optical neuromodulation methods lack cell-type specificity, and all neurons within the range of illumination are theoretically subject to the effects thereof. In the early 2000s, the advances of genetics and molecular biology enabled selective photostimulation of genetically modified neurons. Specifically, the photo-uncaging of ligands that bind to ionotropic receptors provides a means to optically stimulate genetically modified neurons that ectopically express these receptors [4,5]. Finally, optogenetics combines many desirable advantages of neuromodulation with light: the illumination can be spatially confined to the diffraction limit; neural activity can be modulated with the temporal resolution of single spikes; and activation is genetically targeted via the expression of opsins in neurons that normally lack them [6,7].

All methods used in biology are limited by their physical constraints. For optogenetics, delivery of light in neural tissues represents one of the biggest challenges, deeply rooted in the intrinsic interaction between light and matter. The brain is a highly scattering and absorbing medium, owing to the presence of lipids in a wide range of size scales (e.g. neuron membranes and the myelin sheath) in an aqueous environment, and endogenous chromophores (e.g. heme and dopamine). To address this challenge, many methods have been developed to deliver light precisely to the targeted region in the nervous system with minimal acute and chronic damage to the tissue and reduced restraint of the animal's native behavior. The motivation behind these different approaches also represents the main incentive for this review paper.

In this review, we present an overview of the latest optical approaches for neuromodulation with a focus on strategies to deliver light *in vivo* with reduced invasiveness and restraint of the subject. Specifically, we begin with a theoretical framework of the physical principles involved in the development of these methods, citing relevant mathematical formulations to facilitate the understanding of physics constraints in these methods. We then present advances in optical neuromodulation in seven modules: conventional optical fibers, multifunctional fibers, optical waveguides, light-emitting diodes (LEDs), upconversion nanoparticles, optical neuromodulation based on the secondary effects of light, and unconventional light sources facilitated by ultrasound and magnetic fields. We end this review paper by providing our perspective on this field based on the aforementioned physical principles and recent developments in related fields.

## PHYSICAL PRINCIPLES BEHIND OPTICAL NEUROMODULATION METHODS

A more complete theoretical framework of physical principles related to optical neuromodulation methods is available in the Supplementary Data.

### Physical representations of light propagation

Understanding the physical constraints of optical neuromodulation methods requires fundamental knowledge in the electromagnetic theory of light propagation and light–matter interactions. To this end, we can express light propagation in one dimension (1D) in terms of the instantaneous electric field $\vec{E}$ as a function of position *z* and time *t*:

(1) \begin{equation*} {\rm{\ }}\vec{E} = \overrightarrow {{E_0}} \ {\rm exp}\left( { - k^{\prime\prime}z} \right){\rm exp}\left( {ik^{\prime} z - i\omega t} \right), \end{equation*}

where $\overrightarrow {{E_0}} $ is the amplitude of the electric field in the propagating wave and ω is the angular frequency, while $k^{\prime}$ and $k^{\prime\prime} $ are the real and imaginary parts of the complex wavevector, $k$, in scalar representation. Here, $k^{\prime}$ contains the information of the phase velocity of light propagation, and as a result, the refractive index (RI) of the medium. In addition, $k^{\prime\prime}$ reflects the absorption of light in the medium. Based on Equation 1, we can express light intensity, which is defined as the magnitude of the Poynting vector, as follows:

(2) \begin{equation*} I\ = \frac{1}{2}\ {\mathop{\rm Re}\nolimits} \left\{ {\sqrt {\frac{\varepsilon }{\mu }} } \right\}{\left| {\overrightarrow {{E_0}} } \right|^2}\exp \left( { - \frac{{4\pi n^{\prime\prime}z}}{\lambda }} \right), \end{equation*}

in which λ is the wavelength of light, $\varepsilon $ and μ are the permittivity and permeability of the brain tissue, respectively, and $n^{\prime\prime}$ is the complex RI that indicates the absorptivity of the brain tissue. Light intensity should have a dimension of energy per unit area and time (i.e. J m^–2^ s^–1^ or W m^–2^ in SI units).

### Scattering of light in brain tissue

Scattering represents a common behavior of light–tissue interaction in the brain that contributes to the attenuation of light intensity over depth. Specifically, scattering of light in a medium is due to the heterogeneity of the RI in the medium. For light–brain interactions, this heterogeneity usually occurs on the level of nanometers to microns as the result of the spatial separation of water and lipid molecules with distinct RIs [8]. For example, a myelinated axon comprises an aqueous core with an RI of ca. 1.36 and an optically dense sheath with an RI of ca. 1.44 [9]. In this example, the myelin sheaths act as scatterers in an aqueous medium, much akin to oil droplets in the aqueous phase of milk, thus effectively blocking the transmission of light through the brain.

The scattering (and absorption) of spherical particles in a medium can be solved mathematically by the Mie theory to yield the scattering cross section, *C*_sca_. The scattering cross section is defined as the ratio of the power of scattering to the incident light intensity, with the exact solution as well as the Rayleigh approximation available in the Supplementary Data. Understanding the scattering of brain tissue requires knowing both the scattering cross section (how strongly each scatterer scatters light) and the density of scatterers (how many scatterers there are). The scattering coefficient μ_s_ is thus defined as the product of the density of scatterers, ${\rho _s}$, and C_sca_:

(3) \begin{equation*} {\rm{\ }}{\mu _s} = {\rho _s}\ {C_{sca}}. \end{equation*}

**Figure 1.**
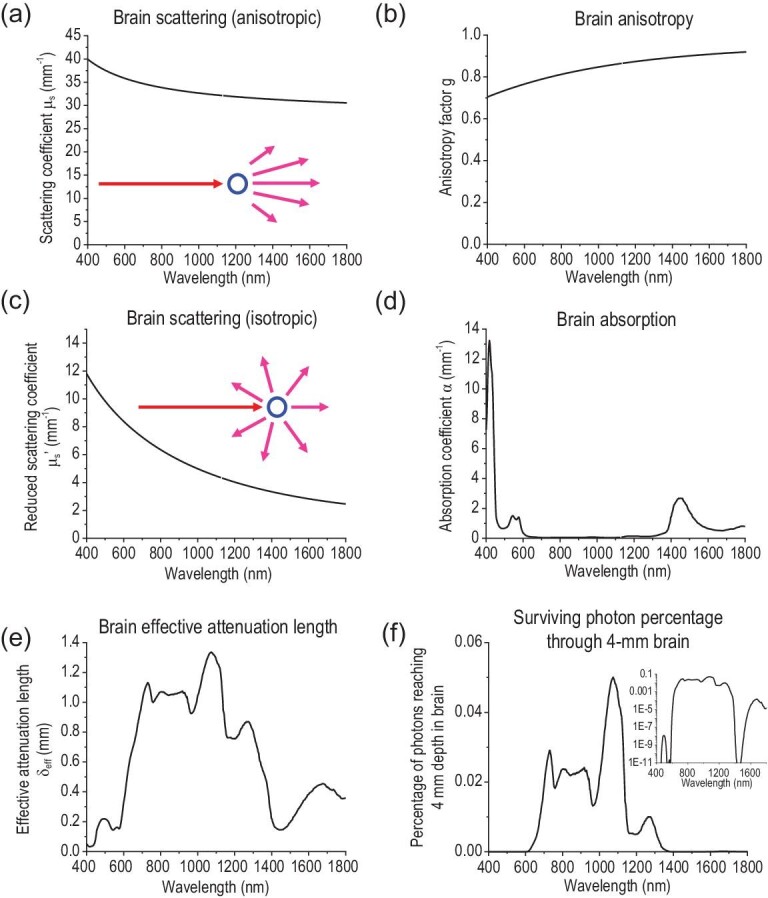
Wavelength-dependent optical properties of the brain. The (a) scattering coefficient μ_s_, (b) anisotropy factor g, (c) reduced scattering coefficient μ_s_’, (d) absorption coefficient α, (e) effective attenuation length and (f) the percentage of photons surviving 4-mm brain tissue are plotted as a function of wavelength in the 400–1800 nm optical spectrum. The insets in (a) and (c) indicate anisotropic and isotropic scattering, respectively. The inset in (f) represents the same graph plotted in logarithmic scale. The data in (a–c) are from ref. [24], the data in (d) are reproduced from ref. [19] and the data in (e) and (f) are calculated from Equations 7 and 9, respectively.

We can interpret μ_s_ as the inverse of the scattering mean free path, i.e. the average distance between two consecutive scattering events. Therefore, light experiences more scattering events within the same path length in a more scattering medium with a large μ_s_. Due to fixed ${\rho _s}$ in a specific tissue, the wavelength dependence of μ_s_ reflects that of C_sca_, exhibiting a monotonic decrease with an increasing wavelength (Fig. 1a) [10].

The physical interpretation of μ_s_ in the context of the scattering mean free path provides a handy method for modeling scattering with the Monte Carlo method, which simulates a random walk of photon packets through three-dimensional (3D) space [11]. Due to the anisotropy of scattering, photon packets ‘remember’ their original travel direction after each scattering event (Fig. 1a, inset). The scattering coefficient μ_s_ and anisotropy factor g (Fig. 1b) can be combined to yield a new quantity, the reduced scattering coefficient, μ_s_’:

(4) \begin{equation*} {\rm{\ }}\mu _s^{\prime} = {\mu _s}\ \left( {1 - g} \right). \end{equation*}

Therefore, μ^′^_s_ describes the diffusion of photon packets in a completely random walk with no ‘memory’ of the previous direction of propagation after scattering. The inverse of μ^′^_s_ is the transport mean free path, the traveled distance of a photon packet after which the ‘memory’ of the original propagation direction is eliminated (Fig. 1c, inset). Similar to μ_s_, μ^′^_s_ also decreases with an increasing λ (Fig. 1c).

### Absorption of light in brain tissue

Besides scattering, absorption represents another mechanism contributing to the attenuation of light in brain tissue. One can define the absorption coefficient α as follows:

(5) \begin{equation*} \alpha \ = \frac{{4\pi n^{\prime\prime}}}{\lambda }. \end{equation*}

With this definition, Equation 2 can be reduced to a more familiar form:

(6) \begin{equation*} I\ = {I_0}\ {\rm exp}\left( { - \alpha z} \right), \end{equation*}

where *I*_0_ combines all pre-exponential terms. The wavelength dependence of α of the brain tissue is shown in Fig. 1d. As can be seen, the absorption spectrum of the brain has two prominent features: first, the absorption peaks below 600 nm are attributed to hemoglobin in the blood; second, several absorption peaks at 980 nm, 1200 nm and 1450 nm correspond to the overtones of water molecules, which are abundant in all soft biological tissues [12]. Apparently, specific brain regions may exhibit distinct absorption properties from the bulk brain due to the accumulation of certain chromophores. For example, neuromelanin gives rise to the black color of the substantia nigra (literally ‘black substance’ in Latin) in the brain, due to a broad absorption spectrum extending from the ultraviolet (UV) to the near-infrared (NIR) region [13]. Therefore, it is necessary to consider the heterogeneity of light absorption in brain tissue when designing optical neuromodulation methods targeting different brain regions.

A direct consequence of light absorption is the elevated temperature in the local brain region. Stujenske and Gordon *et al*. modeled light-induced heating in brain tissue for optogenetics. Due to the strong absorption of 532-nm light in the brain (Fig. 1d), their model predicts a gradual temperature increase up to 2.2°C within a few hundred microns of the light source at 10 mW output (e.g. an optical fiber) [14]. Owen and Kreitzer *et al*. later found that this temperature increase alone can activate an inwardly rectifying potassium channel in the striatum, thus biasing behavioral results of optogenetic studies [15]. Since a threshold intensity is usually required for opsin activation (e.g. 1 mW/mm^2^ for channelrhodopsin-2 (ChR2) and 10 mW/mm^2^ for halorhodopsin (NpHR)) [16,17] and since long periods of continuous illumination are commonly used for optogenetic inhibition, it is crucial to control for non-specific heating due to tissue absorption in optical neuromodulation methods [18].

### Penetration depth of light in brain tissue

The discussion of scattering and absorption above lays the foundation for understanding the penetration depth of light in brain tissue. The effective penetration depth of incident light is given as:

(7) \begin{equation*} {\rm{\ }}{\delta _{eff}} = \frac{1}{{\sqrt {3{\mu _a}\left( {{\mu _a} + {\mu _s}^{\prime}} \right)} }}.\ \end{equation*}

The effective penetration depth of light in the brain in the range of 400 to 1800 nm is plotted in Fig. 1e. Furthermore, the inverse of the effective penetration depth is usually referred to as the effective attenuation coefficient:

(8) \begin{equation*} {\rm{\ }}{\mu _{eff}} = \frac{1}{{{\delta _{eff}}}}\ = \sqrt {3{\mu _a}\left( {{\mu _a} + {\mu _s}^{\prime}} \right)}.\ \end{equation*}

Thus, the incident light intensity decays exponentially with depth as follows:

(9) \begin{equation*} I\ = {I_0}\ {\rm exp}( { - {\mu _{eff}}z} ). \end{equation*}

It is noteworthy that the maximum penetration depth is found at 1070 nm, which is close to the operation wavelength of the Nd:YAG laser, 1064 nm. The wide availability of the Nd:YAG laser and the maximum penetration depth of light near its operation wavelength in the brain thus make this wavelength region (1050–1100 nm) attractive for deep-brain neuromodulation [19]. As a comparison, we estimate the percentage of photons reaching a depth of 4 mm in the brain in Fig. 1f. Specifically, we choose four representative wavelengths relevant in optogenetics: 470 nm, which corresponds to the maximum activation of ChR2; 635 nm, which is the activation wavelength of opsins with the greatest red shift to date [20,21]; 980 nm, which is the wavelength used for deep-brain optogenetics with upconversion nanoparticles [22,23]; and 1064 nm, which is a representative NIR wavelength at which the Nd:YAG laser operates. As expected, 1064-nm light attenuates by ∼20 fold over a depth of 4 mm, in stark contrast to the attenuation of 10^9^ fold at 470 nm, 10^3^ fold at 635 nm and 10^2^ fold at 980 nm over the same depth. The attenuation of light at different wavelengths is derived from the spectral properties of brain tissue reported in ref. [19] and [24].

### Optical waveguides for light delivery

The attenuation of light in the brain, which represents a combination of both scattering and absorption, prohibits efficient light delivery into the deep brain regions. To address this challenge, optical waveguides, such as optical fibers and dielectric slab waveguides, are commonly used for illuminating a specific brain region with efficient light delivery. A good optical waveguiding material should exhibit minimal attenuation of light traveling therein. In addition, propagating light must be spatially confined within the optical waveguide by satisfying the following inequality for an optical fiber:

(10) \begin{equation*} {n_1} > {n_2}, \end{equation*}

where n_1_ and n_2_ represent the RI of the core and cladding material of the fiber, respectively. We will see how the inequality of (Equation 10) is satisfied in a number of fiber optic devices for optogenetics later.

Besides optical fibers, the planar slab waveguide confines propagating light in the waveguiding medium with an RI of n_g_ between the substrate (n_s_) and the cover (n_c_). The longitudinal wave vector, $\beta $, which is the component of wave vector *k* in the propagating direction, needs to satisfy the following inequalities:

(11) \begin{equation*} {\rm kmax}({n_s},{n_c})\ < \ \beta \ < \ k{n_g}. \end{equation*}

## OPTICAL NEUROMODULATION TECHNIQUES

### Conventional optical-fiber-based optogenetic interfaces

Implementation of optogenetics *in vivo* requires delivery of light into the brain of live animals, which is subject to the physics of light–tissue interaction discussed above [25]. Specifically, neurons that are genetically modified with light-sensitive proteins (i.e. opsins), such as ChR2 or NpHR, can be excited or inhibited by blue light (∼470 nm) or yellow light (∼590 nm), respectively. To deliver and express opsin genes, three methods are primarily used: viral transduction, transgenic or knock-in mouse lines and Cre recombination [26]. Specifically, genetically engineered viruses, such as lentiviruses (LVs) and adeno-associated viruses (AAVs), offer fast and efficient expression with high spatial localization [26]. Compared to LVs with larger sizes, AAVs can be packaged into higher titers, thus exhibiting a higher expression level with a larger volume of transduced tissue [27]. Various serotypes of AAVs feature distinguished transduction characteristics in terms of the transduced cell type, transduction level and spatial spread [28]. For example, AAV1 and AAV5 provide a widespread neuronal expression while the expression of AAV2 is more confined in space, with a 5- to 8-fold smaller transduced volume in the hippocampus region [29]. After successful opsin expression, light sources are applied to illuminate the genetically modified neurons.

To deliver light to the superficial regions of the brain, LEDs can be placed on the skull or a cranial window [30]. Despite the success in modulating cortical activity, the strong scattering and absorption of visible light in biological tissue makes it challenging to deliver light to deep-brain regions of interest without a transmitting device or agent. This challenge is visualized quantitatively in Fig. 1e, which reveals a 1/e decay distance of merely 0.1 mm for 470-nm incident light. To this end, low-loss optical fibers are commonly implanted into the brain to create light paths for *in vivo* optical stimulation in an effective and precise manner.

**Figure 2.**
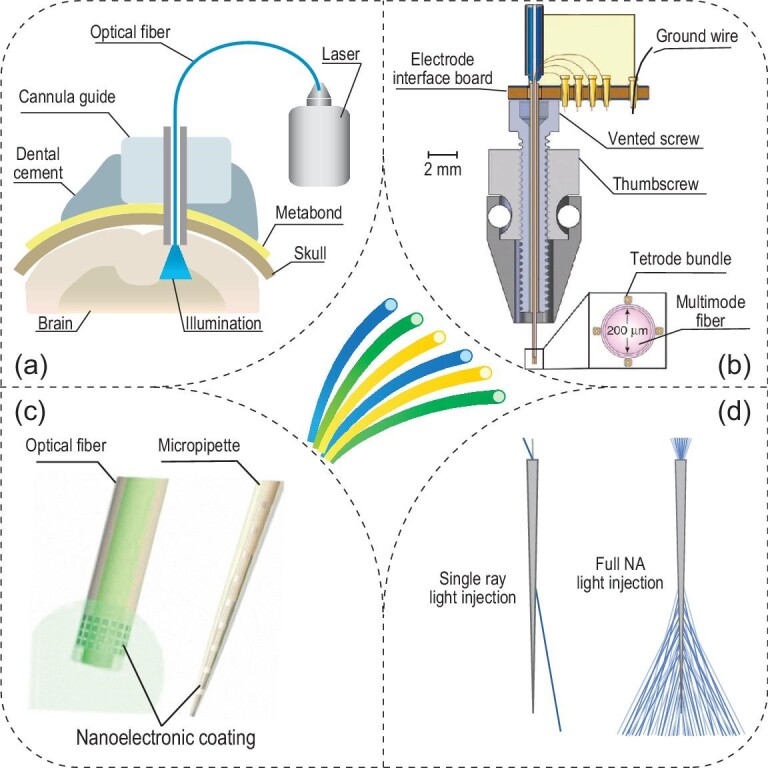
Optogenetic interfaces based on conventional fibers and their derivatives. (a) A diagram of the conventional optogenetic interface based on an implanted optical fiber. Adapted with permission from [32]. Copyright 2010, Nature Publishing Group. (b) A vertical cross-sectional schematic of the optetrode. Adapted with permission from [35]. Copyright 2011, Nature Publishing Group. (c) Schematics of two nanoelectronic coatings that result in multifunctional probes. Adapted with permission from [37]. Copyright 2017, American Chemical Society. (d) Tapered optical fibers enable the illuminated volume to be adjusted based on the numerical aperture of light injection. Adapted with permission from [38]. Copyright 2017, Nature Publishing Group.

The earliest fiber-optic neural interface was implemented by Aravanis *et al*. [31] and was later presented in more detail by Zhang *et al*. (Fig. 2a) [32]. In general, a cannula is used to guide the insertion of the optical fiber to a specific brain target that harbors genetically modified neurons. This very cannula can also be used as a guide to deliver viral vectors into specific brain regions. Besides light intensity measurements at the tip of the fiber, activity measurements of light-sensitive neurons upon illumination are commonly used to validate successful optogenetic excitation or inhibition [32]. The most straightforward method of neuron activity measurements is via simultaneous electrical recordings of neural activities. To this end, metal microwires have been manually attached to optical fibers to realize a bidirectional interface between the implanted device and the subject [33,34].

To validate the capability of optogenetics in regulating neural circuit activity *in vivo*, Anikeeva *et al*. developed a high-throughput electrophysiology platform coupled with optical fibers for simultaneous optogenetics and extracellular neural recordings of light-modulated firing dynamics (Fig. 2b) [35]. In this method, four tetrode bundles composed of a total of 16 polymer-coated NiCr microwires are attached to the shaft of an optical fiber, which acts as both a light source and a structural support for the tetrodes. This assembly thus yields a multichannel device termed the ‘optetrode’. Since the optetrode enables single-unit electrophysiological recordings from distinct locations surrounding the central optical fiber, diverse brain activity patterns are observed under different illumination conditions. The utility of the optetrode in behavioral neuroscience experiments has been confirmed in ChR2-expressing and non-expressing animals engaged in an open field exploration task.

In the optetrode, rigid metal wires exacerbate the mechanical mismatch between the implant and brain tissue, usually leading to chronic tissue damage and an elevated immune response, such as neuron death, glial scarring and astrocyte reactivity [36]. To mitigate these challenges and retain the multichannel recording capability in a fiber-optic system, nanoelectronic coating (NEC) with a thickness of <1 μm has been conformally applied to the surface of the traditional optical fiber, forming a multifunctional neural probe with a diameter of 30 μm (Fig. 2c) [37]. Assisted by surface tension, NEC devices naturally wrap around a curved hosting probe when they are lifted across the water–air interface. The utility of these devices is validated by simultaneous recordings and optogenetic stimulation in the medial prefrontal cortex (mPFC).

Another challenge of the conventional optical fiber arises from its limited illumination volume, which is usually confined to the tip of the fiber. To this end, tapered optical fibers have been engineered to control and adjust their illumination volumes inside the brain (Fig. 2d) [38]. The tapered design permits the selection of optical modes and reduces tissue damage. The focused ion beam (FIB) micromachining technique enables the creation of optical windows along the tapered region, while gold plating is used to prevent light leakage [39]. To examine the illumination profile, simulations, and benchtop and *in vivo* animal experiments, are conducted under various incident light angles and with different numbers of optical windows. Coupled with a Neuronexus silicon probe, tapered fibers demonstrate the reconfiguration of light delivery at multiple points with immediate neural responses. In a later study, large volumes of brain illumination were accomplished by full numerical aperture (NA) light injection through the tapered fiber, while restricted light illumination could be quickly adjusted by modifying the incident angle of a single input ray [38]. The authors observed divergent behavioral outcomes from the animals when different brain regions were stimulated via a single implanted tapered fiber, thus validating its unique advantages for optogenetics.

Besides new advances in optical fibers, engineered opsins have been developed to afford red-shifted activation spectra [40–42] and ultra-sensitive light responses [21,43,44], thus enabling transcranial optogenetics with optical fibers mounted on the skull without invasive implantation in the brain. A recent demonstration by the Deisseroth lab leverages the high sensitivity and red-shifted activation of the potent channelrhodospin, ChRmine, to achieve transcranial modulation of deep-brain neural circuits. Combined with systemic viral delivery [45], ChRmine enables neuromodulation of behaving mice without any intracranial surgery. A significant advantage of 635-nm responsive ChRmine over 470-nm responsive ChR2 arises from the much greater brain penetration of 635-nm photons than 470-nm photons. Specifically, Fig. 1f reveals that with the same incident photon flux, a million times more 635-nm photons can penetrate through 4-mm brain tissue than their 470-nm counterparts. However, it is worth noting that only 0.1% of 635-nm photons incident on the brain surface can penetrate to a depth of 4 mm. Given the wavelength-dependent penetration depth shown in Fig. 1e, we envision that opsins responsive to longer-wavelength NIR light may offer transcranial and transdermal deep-brain optogenetics with the greatest penetration and least non-specific heating.

### Multifunctional fibers as versatile optical interfaces

In the previous section, outfitting conventional optical fibers with existing recording electrode technologies permits a straightforward solution for simultaneous optogenetics and electrophysiology *in vivo*. However, the large footprint, mechanical rigidity and complicated assembly procedure prevent them from achieving optical manipulation and electrical recordings in long-term neuroscience studies. Micropipette pulling, which represents one of the most classical techniques in neuroscience, offers an elegant strategy for miniaturizing the optical fiber while integrating multiple functions therein [46]. Using a conventional micropipette puller, the De Koninck lab fabricated a dual-core microprobe with a graded index optical fiber for light transmission and a hollow core filled with an electrolyte solution (e.g. 1–3 M NaCl) for electrical recordings [46]. Despite the integrated optical and electrical modalities in the same microprobe, this platform only harbors a single electrode and thus disallows multiplexed recordings.

To address the challenges of conventional optical fibers, the Anikeeva lab has pioneered the development of a series of flexible, multifunctional and biocompatible fibers [47,48]. Unlike the micropipette pulling method, the thermal drawing process (TDP), which is conventionally used for fabricating optical fibers, is employed to incorporate a wide array of functional elements. These functional elements include recording electrodes of different materials (e.g. conductive polyethylene and tin) and diameters, optical waveguides with low loss (1.6–2.6 dB/cm) and microfluidic channels, all incorporated in a single device. In a typical TDP, a bulky template, the ‘preform’, is fabricated via a standard machining and consolidation process, after which hundreds of meters of long thin fibers can be drawn under an applied stress and an elevated temperature. It is noteworthy that only a few thermoplastics or low-melting-point materials comply with the drawing temperature of the surrounding polymer matrix for making the embedded polymer waveguides or electrodes [49]. Specifically, for fabricating an all-polymer waveguide with different refractive indices, polycarbonate (PC) with an RI of 1.58 serves as the core material, while the cyclic olefin copolymer (COC; RI = 1.53) [47], poly(methyl methacrylate) (PMMA; RI = 1.48) [50] or polyvinylidene difluoride (PVDF; RI = 1.426) [51] are used as its pairing cladding material. Note the general requirement of ${n_{core}} > {n_{cladding}}$, which is mathematically derived in (Equation 10) above. Additionally, the convergence-fiber drawing method makes it possible to incorporate silica fibers inside the multifunctional fiber probes, thus mitigating the transmission loss in polymer waveguides [52].

**Figure 3.**
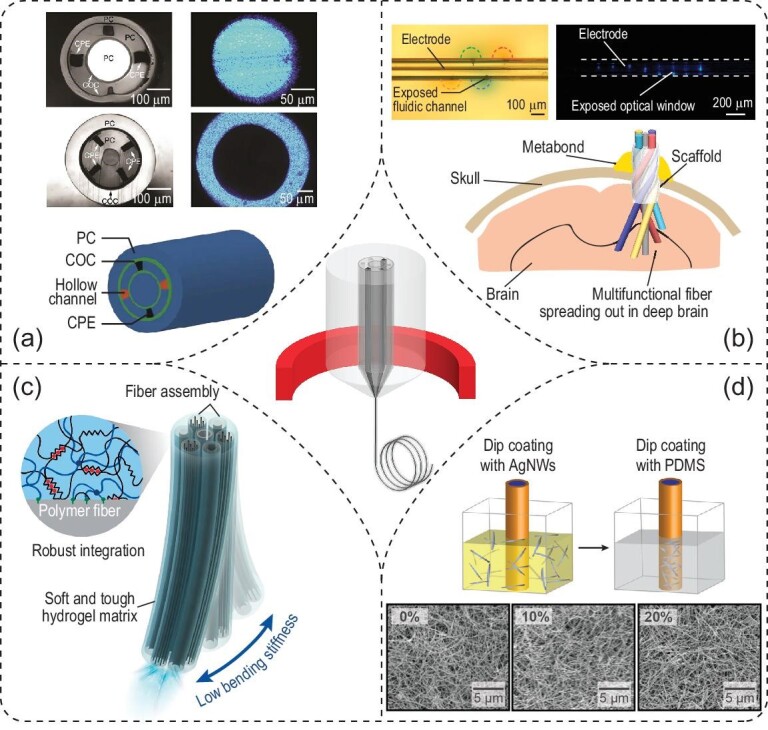
Optogenetic interfaces based on multifunctional fibers. (a) Cross-sectional photographs (top panels) and schematic (bottom panel) of multifunctional fibers. Photographs in the right column reveal light propagation in the core and outer layer of two different multifunctional fibers. Adapted with permission from [47]. Copyright 2015, Nature Publishing Group. (b) Depth-dependent (top panels) and spatially expandable (bottom panel) multifunctional fibers enable interfacing with a larger volume of the neural tissue. Adapted with permission from [51]. Copyright 2020, Nature Publishing Group. (c) Adaptive hydrogel hybrid probe incorporates fiber probes of different functions in a hydrogel matrix. Adapted with permission from [53]. Copyright 2021, Nature Publishing Group. (d) Stretchable multifunctional fibers. Top: the dip-coating process to fabricate the stretchable electrode composed of AgNWs. Bottom: scanning electron microscopy (SEM) images of the stretchable electrode reveal similar connectivity in the mesh structure under different strains. Adapted with permission from [54]. Copyright 2017, American Association for the Advancement of Science.

In the first demonstration of multifunctional fibers, optical waveguides were realized in both the core and ring structures via the RI contrast between PC and COC. Also present in the multifunctional fibers were electrodes composed of a conductive polymer composite (e.g. conductive polyethylene (CPE)) and microfluidic channels (Fig. 3a) [47]. To validate the multifunctionality of this device, the authors performed electrophysiological monitoring of neural activities and simultaneous optogenetics stimulation in the mPFC of freely moving, transgenic Thy1-ChR2-YFP mice. In addition, the authors leveraged the built-in microfluidic channels to inject synaptic blockers, 6-cyano-7-nitroquinoxaline-2,3-dione (CNQX), into the same brain region. As a result, the optically evoked neural activities were significantly diminished despite light stimulation, followed by slow recovery and restoration of original firing patterns. A salient advantage of multifunctional fiber arises from its reduced dimensions and stiffness, making it comparable to human hair, thus leading to a minimal glial response and the long-term recording capability (over two months after implantation) of single units.

The demonstrated versatility of multifunctional fibers also enables one-step optogenetics [48]. Traditional optogenetic experiments in wild-type mice require a two-step surgery, in which the first surgical step delivers viral vectors for gene transduction, while the second step allows for the implantation of optical fibers or optrodes [32]. This traditional approach, also commonly practiced, imposes repeated tissue damage and often results in the misalignment between transgene delivery and activation. In contrast, multifunctional fibers offer an elegant one-step alternative with lower invasiveness and greater precision. Specifically, the built-in microfluidic channels enable the delivery of viral vectors containing opsin genes to the targeted brain region in the wild-type mouse brain. In addition, the neighboring electrodes and optical waveguide, which are constructed in the same device to naturally ensure the alignment, offer time-dependent monitoring of opsin expression over weeks. Using these devices implanted into multiple locations in the mouse brain, the authors demonstrated opto-electrophysiological studies of projections from the basolateral amygdala (BLA) to the mPFC and ventral hippocampus (vHPC) in behaving animals.

One common challenge in fiber-based optogenetics arises from the limited volume of illumination in the brain. Nonetheless, the intrinsic flexibility of multifunctional fibers enables an elegant solution to address this issue. The Jia group recently invented a fiber-based spatially expandable neural interface (Fig. 3b) [51]. In this approach, a femtosecond laser micromachining technique is used to first create a depth-dependent fiber probe with exposed functional interfacing sites along the fiber length, thus resulting in a layout similar to a single-shank Michigan probe. In addition, a spatially expandable fiber probe is realized by leveraging a unique helical scaffolding fiber to afford 3D coverage in space. After implantation, both depth-dependent and spatially expandable fiber probes enable optogenetic neuromodulation with simultaneous electrical recording, with chronic stability over five months. Specifically, these implanted fiber probes can record neural activity from various brain regions, such as the cortex, CA1 and CA3 fields of the HPC, hypothalamus, thalamus and amygdala in a mouse model of infection-induced epilepsy.

Another challenge of multifunctional fibers arises from the trade-off between the need to incorporate multiple functional elements and the inevitable increase in their bending stiffness. To mitigate this challenge, an adaptive hydrogel hybrid probe has been realized by encapsulating a bundle of fibers with different functions in the hydrogel matrix (Fig. 3c) [53]. Specifically, UV irradiation bonds the pre-gel solution to the surface of functional fibers via a one-step polymerization process, resulting in a thin and uniform hydrogel layer covering the fiber array. This probe has at least two advantages: first, the initial bending stiffness of the probe ensures deterministic implantation into deep brain regions. Second, hydration and swelling of the hydrogel coating after implantation results in a tissue-favorable matrix, leading to a substantial decrease in observed foreign body responses. As a result, the adaptive hydrogel hybrid probe exhibits stable performance in recording optically evoked potentials and single units for up to six months, and a reduced immune response.

Other than applications in the brain, multifunctional fibers have also been used in the spinal cord (Fig. 3d) [54]. To accommodate the deformation of the spinal cord during the natural movement of the subject, stretchable COC elastomer (COCE; RI = 1.51) and polydimethylsiloxane (PDMS; RI = 1.41∼1.47) were chosen as the core and cladding material, respectively. Silver nanowires (AgNWs) were dip-coated onto the surface of the fiber to form a conductive ring electrode, maintaining good electrical conductivity under applied strains. Electrical recordings of the spinal cord with optical stimulation demonstrated the suitability of stretchable fiber probes for studying the peripheral nervous system (PNS) [55,56]. More recently, stretchable optical fibers consisting of styrene-ethylene-butylene-styrene (SEBS) with different refractive indices and helical stretchable polymer electrodes have been reported with multi-axial sensing modalities under extreme deformation conditions [57].

### Optical waveguides for individually addressed and multiplexed optogenetics

**Figure 4.**
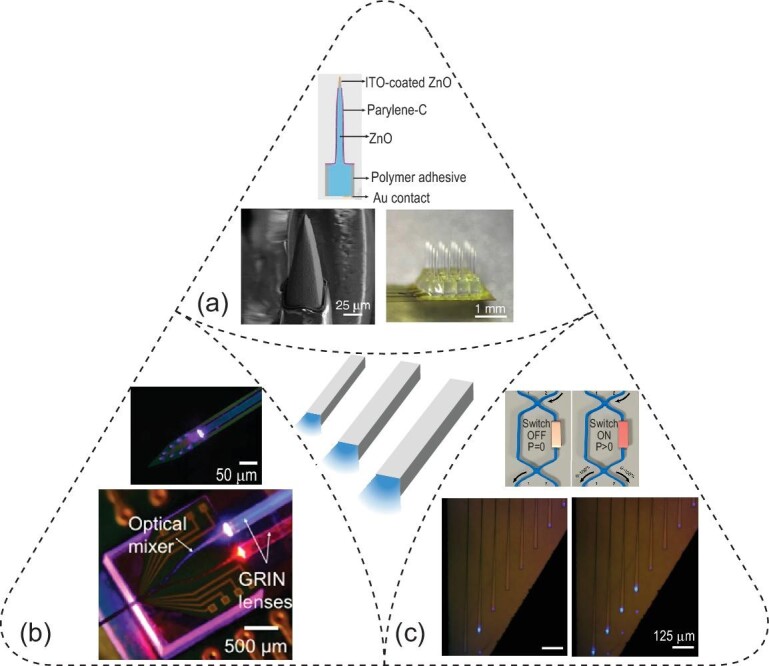
Optogenetic interfaces based on multiplexed waveguides. (a) A micro-optoelectrode array (MOA, bottom right) based on optically transparent and electrically conductive ZnO pillars (top) with chemically etched and ITO-coated tip (bottom left). Adapted with permission from [58]. Copyright 2015, Nature Publishing Group. (b) Multicolor photonic circuit in a Michigan-probe configuration (top), enabling optical coupling and mixing via GRIN lenses and an optical mixer, respectively. Adapted with permission from [60]. Copyright 2016, Nature Publishing Group. (c) Reconfigurable nanophotonic silicon probe based on multimode interferometer (MMI)-based switches (top), enabling individually addressable light output at desired illumination ports (bottom). Adapted with permission from [62]. Copyright 2020, Nature Publishing Group.

In the previous section, different materials are used for optical waveguiding and electrical recordings in separate channels. In contrast, some materials afford simultaneous electrical conductivity and optical transparency, thus affording multiplexed waveguiding and recordings through the same channels. For instance, a 4×4 ZnO-based micro-optoelectrode array (MOA) with a geometry of a Utah array was created for simultaneous and multiplexed recordings and optogenetic stimulation (Fig. 4a) [58]. Specifically, ZnO pillars (RI ∼2.0) coated with parylene-C (RI ∼1.6) acted as both the waveguide and the recording electrode. Multiplexity in optical stimulation was realized via a laser-scanning system, which spatially controlled light input to individual optoelectrodes with tunable light intensity during *in-vivo* optogenetic studies. Finally, multiplexed optical modulation with simultaneous recordings revealed the causal relationship between cortical microcircuit dynamics and evoked limb motions in anesthetized mice.

The back-end optical interfacing used in the MOA required a customized laser-scanning system, thus presenting a technical challenge for its wide adoption. Hence, a monolithically fabricated photonic circuit offers an alternative approach, with efficient input light coupling and compact packaging. The first reported on-chip waveguide measures 11 μm × 20 μm at the distal end and is composed of silicon oxynitride (RI = 1.51) as the core material and silicon dioxide (RI = 1.46) as the cladding. The waveguide assumes the layout of a typical Michigan probe and can be coupled to a laser via an optical fiber [59]. A more compact design with fiberless coupling is accomplished by coupling the waveguide mixer to the side-emitting injection laser diode via a gradient-index (GRIN) lens (Fig. 4b) [60]. Leveraging micropatterned photonic circuits, blue and red light can be directed into a shared waveguide port for optically modulating the same population of pyramidal cells in the mouse brain, that expresses both ChR2 and eArch3. Four of these multicolor optoelectrodes can be further packaged in parallel into a multi-shank optogenetic probe for dissecting complex neural circuits [61].

Although the integration of waveguides with electrodes into a chip-scale device creates new opportunities, the multiplexity of light-emitting sites remains limited. To overcome this challenge, a silicon-based neural probe harboring nanophotonic circuits has been developed to achieve multiple reconfigurable optical paths after implantation (Fig. 4c) [62]. In this device, optical waveguides with a cross-sectional area of 200 nm × 350 nm are made of a SiN core (RI = 1.996) and a SiO_2_ cladding (RI = 1.446). To afford tunable configuration, a waveguide mixer is equipped with a localized microheater, acting as a switch, which can tune the RI of the mixer via a local temperature increase. These multimode interferometer (MMI)-based switches can thus steer the direction of light propagation into predefined optical output ports. Furthermore, full integration of nanophotonic and nanoelectronic circuits allows recordings via Pt electrodes, thus suggesting the scalability and complementary metal-oxide-semiconductor (CMOS)-compatibility of this technology.

### Wired and wireless LED devices for optogenetics

**Figure 5.**
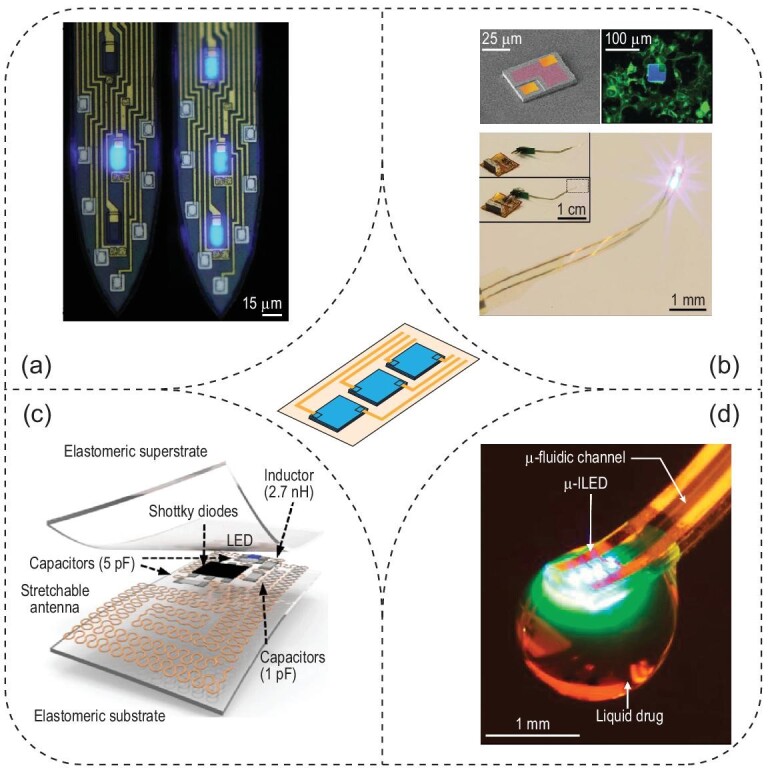
Optogenetic interfaces based on wired and wireless LED devices. (a) Wired μLEDs integrated in multi-shank Michigan probes. Spatially addressable illumination patterns are shown in these two shanks. Adapted with permission from [63]. Copyright 2015, Elsevier Inc. (b) Injectable, cellular-scale optoelectronic device for wireless optogenetics. Top left: a colorized SEM image of the μ-ILED. Top right: a fluorescence image of the μ-ILED with yellow fluorescent protein (YFP, green)-expressing human embryonic kidney (HEK) cells. Bottom: wirelessly powered integrated system with light emission. Adapted with permission from [67]. Copyright 2013, American Association for the Advancement of Science. (c) Wirelessly powered LED device in a stretchable elastomeric substrate. Adapted with permission from [70]. Copyright 2015, Nature Publishing Group. (d) Wirelessly controlled μ-ILED and microfluidic channel for simultaneous illumination and drug delivery. Adapted with permission from [74]. Copyright 2015, Elsevier Inc.

Besides light delivery via waveguiding devices, LEDs provide an alternative approach for *in-vivo* optogenetics. One advantage of LEDs arises from their lithographic fabrication, compatible with conventional on-chip input/output (I/O) bonding methods for back-end connection. To this end, Wu *et al*. reported a four-shank silicon probe with each shank containing eight recording sites (Ti/Ir, 11 μm × 13 μm) and three monolithically integrated blue-light-emitting InGaN microscopic LEDs (μLEDs, 10 μm × 15 μm). Owing to photolithographically defined interconnects, all recording electrodes can be independently addressed and all μLEDs independently controlled (Fig. 5a) [63]. Despite these advantages, monolithically fabricated μLEDs exhibited intrinsic artifacts due to electromagnetic interference and photovoltaic effects, which impaired the temporal resolution of recorded neuronal responses to light stimulation [64]. To alleviate these artifacts, a minimal-stimulation-artifact (miniSTAR) μLED was recently developed by introducing a shielding layer to reduce the electromagnetic interference [65]. Moreover, a heavily boron-doped silicon substrate was used to mitigate the photovoltaic effect. In addition, transient pulse-shaping control further diminished lingering stimulation artifacts. Both *in-vitro* and *in-vivo* experiments demonstrated reduced artifacts with miniSTAR μLEDs, thus resulting in high-quality recordings under optogenetic control with high spatiotemporal resolution.

All previous examples, such as optical fibers and wired μLEDs, require a tethered interface for modulated animals. Advanced cable management with optical/electrical commutators has reduced the challenges for studies involving freely moving animals [66]. However, these tethered systems still restrict animal mobility and constrain experimental designs especially in complex and ethologically relevant animal models. To eliminate those constraints, Kim *et al*. developed a tether-free multifunctional optoelectronic system that integrates GaN microscale inorganic LEDs (μ-ILEDs) with Pt microelectrodes, a microscale inorganic photodetector (μ-IPD) and a temperature microsensor or microheater on a removable injection microneedle (Fig. 5b) [67]. The embedded μ-IPD and temperature sensors can provide real-time feedback on the light intensity of the μ-ILEDs and the local temperature changes, respectively. To power the μ-ILEDs, a wireless module was incorporated via a head-mounted printed circuit board for radiofrequency (RF) scavenging. A fully wireless system was implemented in a conditioned place preference (CPP) experiment in a Y maze, where tested animals learned to self-stimulate their dopaminergic neurons by activating the wireless μ-ILEDs. The self-stimulation behavior is a hallmark assay for validating the efficacy of optogenetic interfaces and identifying reward neurons [68].

The externally mounted headstage antenna demands an exposed cranial surface, thus making it challenging to probe the spinal cord or PNS where a stable tissue/skull interface does not exist. Furthermore, the constant movement in the PNS necessitates miniaturized and wireless light sources for optogenetics therein [55]. To address these challenges, a relatively light-weight, wireless and fully internally implantable optogenetic device has been developed and can be powered by a resonant cavity [69]. This device was deployed to three regions of interest, the premotor cortex in the brain, the dorsal horn of the spinal cord and the peripheral nerve ends in the hind paw. The efficacy of this device for optogenetic control was validated by measuring the animals’ circling behavior and running speed as well as quantifying c-fos expression in immunostained tissue slices. Taken together, these experiments demonstrate the utility of wireless μLED devices for optogenetic modulation in the PNS with minimal footprint and reduced invasiveness.

The mechanical mismatch between fully implantable μLED devices and implanted tissue also hinders their compatibility with specific tissue targets, especially in the context of the natural motions of the subject. To reduce the mechanical stress on the spine and sciatic nerve, the Rogers lab developed stretchable wireless LED devices encapsulated in PDMS, which were implanted in rats to improve the dynamic tissue interface (Fig. 5c) [70]. Specifically, the built-in antennas featured a serpentine pattern to harvest RF power with high stretchability. This stretchable design also ensures reliable device activation within a wide bandwidth for RF harvesting since the relevant range of applied strains only induces a relatively small shift in the center frequency. With this device, the authors demonstrated chronic sciatic nerve activation for six months. The utility of this device was tested by studying the spinal pain pathways, in which the device was implanted over the sciatic nerve and the spinal epidural space. Later, a fully implantable and stretchable optogenetic system utilizing a resistive strain gauge was developed by the same group for closed-loop optogenetic control of bladder functions by real-time monitoring and signal processing [71].

While various wireless designs have been created for optogenetics, fine adjustment of the light intensity of μ-ILEDs at multiple illuminating sites still requires full development. To this end, a fully implantable, battery-free optogenetic system was invented, enabling precise regulation of output intensity and independent operation of four spatially distributed light sources [72]. In particular, the integration of multiple active components, such as a purpose-designed rectifier, a microcontroller and a digital-to-analogue converter (DAC), provided a versatile platform for a set of wirelessly powered optogenetic tools. Specifically, the one-way communication protocol allowed for not only individual control of the μ-ILED devices on two bilateral shanks, but also programmable control of multiple devices within a single trial, via a predetermined pulse length combination for each μ-ILED. More recently, an advanced version of this device was applied to flying species such as songbirds [73].

Additional control of neural activities can be achieved by combining the wireless optoelectronic system with pharmacological delivery via integrated microfluidic channels. A compact paradigm of such a multifunctional and wireless platform was presented by Jeong *et al*. with a battery-powered wireless optofluidic neural probe (Fig. 5d) [74]. Notably, each microfluidic channel was connected to a separated reservoir that was programmatically activated for pumping biologics via thermal expansion of gating microspheres induced by Joule heating. Using this optofluidic device, the authors delivered a μ-opioid receptor agonist to modulate the animal's behavior via pharmacological intervention. A real-time place preference test was used to validate this wireless multimodal neural interface: wireless optogenetic activation of the VTA-NAc (ventral tegmental area to nucleus accumbens) projection drove a robust real-time place preference, while wireless delivery of a dopamine receptor D1 antagonist abolished the place preference. Many efforts have since been made towards the optimization of the wireless optofluidic system. For example, an electrochemical micropump was engineered to induce volume expansion by water electrolysis, realizing on-demand fluidic delivery via a wireless and fully implantable optofluidic cuff system [75].

### Upconversion nanoparticles for optogenetics

Besides optical waveguides and light-emitting devices, upconversion nanoparticles (UCNPs) present another mechanism for delivering light into neural tissue for optogenetics. Specifically, upconversion is an anti-Stokes process realized by the absorption of multiple photons of lower energy (longer wavelengths) followed by the emission of a single photon of higher energy (shorter wavelengths). Upconversion luminescence can be generally produced via two processes: excited-state absorption, which is commonly found in lanthanide ions, and triplet-triplet annihilation, which explains upconversion luminescence in some organic chromophores [76]. The advantages of UCNPs can be seen from the wavelength-dependent scattering coefficients in Fig. 1a and c: scattering of light in the brain follows an inverse relationship with wavelength, thus enabling the deeper penetration of NIR light than its visible counterpart.

**Figure 6.**
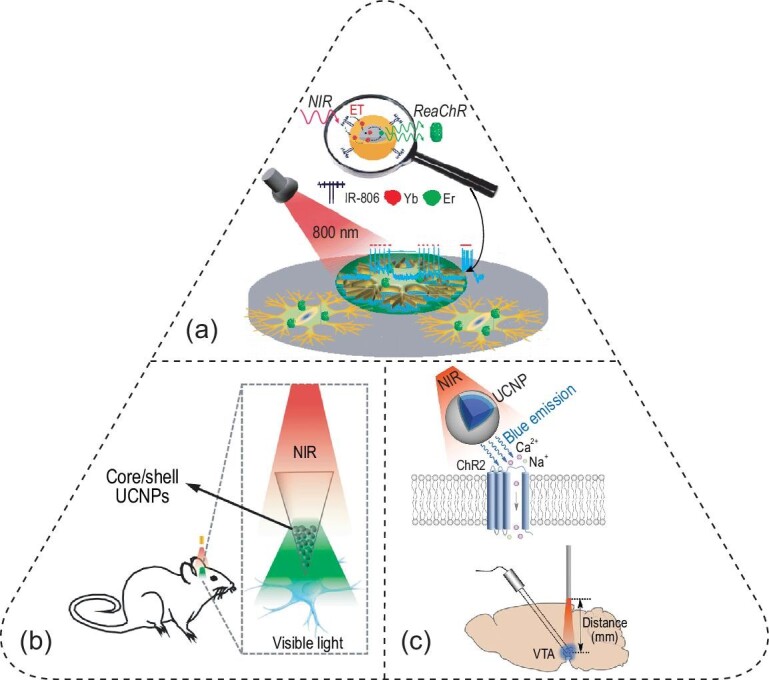
Optogenetics facilitated by UCNPs. (a) NaYF_4_:Yb,Er@NaYF_4_:Yb core-shell nanoparticles convert 800-nm NIR light to 550-nm green emission for activating ReaChR in cultured hippocampal neurons. Adapted with permission from [78]. Copyright 2016, American Chemical Society. (b) An implantable device composed of UCNPs in a glass micro-optrode, converting incident NIR light to visible light emission for *in-vivo* optogenetics. Adapted with permission from [23]. Copyright 2017, Elsevier Inc. (c) NaYF_4_:Yb,Tm@NaYF_4_ UCNPs convert 980-nm NIR light to 470-nm blue emission for activating ChR2 (top), enabling deep-brain optogenetics in the VTA (bottom). Adapted with permission from [22]. Copyright 2018, American Association for the Advancement of Science.

Lanthanide-doped UCNPs offer a unique mechanism to convert low-energy incident NIR light into high-energy visible light of different wavelengths for activating various opsin variants. UCNP-enabled optogenetics was first proposed by Deisseroth and Anikeeva in 2011, followed by its first experimental implementation in 2015. The first demonstration was credited to the Yawo lab, who synthesized NaYF_4_:Sc/Yb/Er UCNPs to convert 975-nm NIR light to 543-nm green light for activating red-shifted channelrhodopsins such as C1V1 and mVChR1 [77]. In another early demonstration, the Han group synthesized dye-sensitized core/active shell UCNPs with sufficient upconversion efficiency, modulating the activity of cultured hippocampal neurons with an 800-nm laser [78]. Under 800-nm irradiation, these UCNPs produced green emission near 550 nm, which activated a red-shifted channelrhodopsin, ReaChR, for modulating the firing activity of cultured hippocampal neurons (Fig. 6a).

Since the advantages of UCNPs lie in their unique ability to convert brain-penetrant NIR light into visible emission, several groups have demonstrated deep-brain optogenetics with either UCNP-containing devices implanted or UCNPs injected into the brain region of interest [22,23,79–81]. Specifically, the Shi group packaged UCNPs in a glass micro-optrode, which acted as an implantable device to convert remotely applied 980-nm NIR light to blue or green light emission from the tip of the micro-optrode in the brain (Fig. 6b) [23]. An impressive accomplishment of this work was the tetherless optical interface enabled by a robotic laser projection system that automatically tracks the animal head during behavioral experiments. The authors also demonstrated multiplexed optogenetic stimulation enabled by doping different ions in their UCNPs. As an example, Tm^3+^ produces 470-nm blue emission for activating ChR2, while Er^3+^ produces 540-nm green emission for activating a red-shifted channelrhodopsin, C1V1 [23,79]. The same group also demonstrated wireless optogenetic inhibition with 980-nm irradiation in the rat brain, which was implanted with a device containing core–shell–shell UCNPs with enhanced emission in the 540–570-nm range [80]. More recently, lanthanide-doped upconversion microparticles were also demonstrated to enable fiberless optogenetics with a 976-nm laser in freely behaving mice [81].

Previous *in-vivo* demonstrations of tetherless optogenetics have been limited to a maximum penetration depth of ca. 2 mm in the rodent brain. This penetration limit is partly owing to the relatively low efficiency of upconversion and the lingering attenuation of NIR light through the brain tissue. To mitigate these challenges, the McHugh and Liu labs optimized the blue-light emission intensity of UCNPs with a core/shell structure comprising a NaYF_4_:Yb,Tm core and an epitaxially grown NaYF_4_ shell with an improved NIR-to-visible conversion efficiency of ∼2.5% (Fig. 6c, top) [22]. Owing to this high efficiency, the authors demonstrated a local 470-nm emission intensity of 0.063 mW/mm^2^ upon an incident 980-nm laser power of 2 W. The authors injected these nanotransducers in the VTA, a deep-brain region located at 4.2 mm underneath the skull (Fig. 6c, bottom). Transcranial delivery of 980-nm light via a skull-fixed optical fiber successfully activated ChR2-expressing dopaminergic neurons, which was validated with fast-scan cyclic voltammetry and c-fos immunostaining. In addition, the authors also demonstrated this approach in multiple neural systems, including successful induction of fear memories in the dentate gyrus of freely behaving mice in a fear-conditioning experiment.

Despite recent advances, several challenges remain for UCNP-based optogenetics. First, upconversion is a multi-photon process with the theoretically maximal quantum yield (QY) of 100/*n*% (*n* = number of absorbed photons). Furthermore, due to the non-linear nature of the upconversion process, the QYs of UCNPs decrease monotonically as the power density goes down, with the QYs <0.1% under physiologically permissible light power densities [82,83]. Second, the excitation of UCNPs is largely fixed at a handful of wavelengths, such as 980 nm and 808 nm, due to the intrinsic electronic structures of lanthanide ions [82]. The limited choices of excitation wavelengths impose challenges for deep-brain optogenetics due to a water absorption peak at 980 nm and lingering scattering at 808 nm [12,84]. For example, the 980-nm wavelength corresponds to a local minimum of penetration efficiency (Fig. 1f), leading to merely 1% of the incident photons surviving 4-mm brain tissue. Based on the wavelength-dependent effective penetration depths in the brain (Fig. 1e), we envision that opsins and nanotransducers responsive to 1064-nm NIR light offer the greatest penetration depth in the brain, thus potentially enabling transcranial and transdermal deep-brain optogenetics throughout the entire rodent brain.

### Photothermal, photoelectrochemical and photoacoustic neuromodulation

Transgene delivery of opsins in neurons represents a challenge for implementing optogenetics in larger mammals, especially humans. In contrast, it has been known for over a century that light, especially high-energy pulsed light, can control the activity of neurons that do not express light-sensitive proteins [1]. Therefore, developing optical methods to modulate the activity of cells without opsin expression provides an alternative to optogenetics for both fundamental studies and clinical applications. To date, many strategies have been applied to achieve optical neuromodulation in the absence of opsins. In particular, four light-based mechanisms are leveraged for neuromodulation: the optocapacitive effect, photothermal activation of transient receptor potential cation channels (TRPs), photoelectrochemical and photovoltaic effects, and mechanical stimulation via the photoacoustic process.

**Figure 7.**
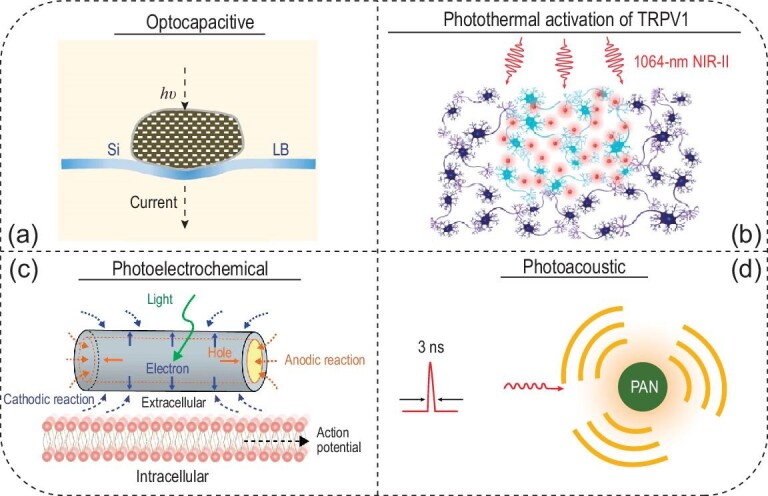
Other optical neural interfaces based on (a) optocapacitive, (b) photothermal, (c) photoelectrochemical and (d) photoacoustic effects. (a) Lipid-supported Si mesostructures convert 532-nm light into transmembrane current to depolarize neurons with high efficiency. Adapted with permission from [85]. Copyright 2016, Nature Publishing Group. (b) Macromolecular infrared nanotransducers for deep-brain stimulation (MINDS) convert 1064-nm NIR-II light into transient local heating for activating TRPV1 channels in the deep mouse brain. This approach eliminates the brain implants and head tethering for neuromodulation in freely behaving animals. (c) Coaxial p-type/intrinsic/n-type (PIN) Si nanowires (SiNWs) produce Faradaic currents to induce action potentials under 532-nm irradiation. Adapted with permission from [97]. Copyright 2018, Nature Publishing Group. (d) Photoacoustic nanotransducers (PANs) convert nanosecond 1030-nm laser pulses into acoustic waves for neuromodulation. Adapted with permission from [100]. Copyright 2020, Elsevier Inc.

The optocapacitive effect requires efficient light absorbers and high photon flux from strong light sources to produce usable energy for exciting cells via the capacitive change of the cell membrane. In this process, a rapid and transient temperature increase occurs upon light irradiation, modulating the capacitance of the cell membrane, which is dependent on the rate of temperature change. This capacitance change in turn induces a capacitive current across the membrane, leading to depolarization of an electrogenic cell such as the neuron (Fig. 7a) [85]. In 2012, Shapiro *et al*. leveraged this mechanism to modulate cultured cells based on the non-specific absorption of water in the infrared [86]. Specifically, water exhibits strong infrared absorption near 1880 nm due to overtone stretching modes [12]. As a result, when a pulsed infrared laser strikes cells cultured in an aqueous environment, the local temperature rapidly increases and drives the capacitive current. In their experiments, the authors demonstrated successful production of capacitive currents induced by transient heating in three models: oocytes, cultured mammalian cells (e.g. human embryonic kidney [HEK] cells) and artificial lipid bilayers, all at varied laser pulse energies.

The abundance of water in biological systems limits the spatial specificity of optocapacitive neuromodulation with lasers of water-absorbing wavelengths. To address this challenge, Carvalho-de-Souza *et al*. leveraged the plasmonic absorption band of gold nanoparticles (AuNPs) near 523 nm, at which wavelength water absorption is minimum. In this work, AuNPs were conjugated to three proteins including a synthetic Ts1 neurotoxin targeting voltage-gated sodium channels, and antibodies targeting TRPV1 and P2X_3_ ion channels, in dorsal root ganglion (DRG) neurons [87]. Conjugated AuNPs exhibited stable photothermal performance, thereby reproducibly evoking action potentials even after convective washout. In addition, mouse hippocampal brain slices were mixed with AuNPs to show the feasibility of optocapacitive neuromodulation in an *ex-vivo* neural tissue model. Facilitated by a voltage-sensitive dye (i.e. indocyanine green (ICG) with infrared fluorescence), the authors validated successful neuromodulation with functionalized AuNPs over a relatively large area in the brain slice.

Owing to their demonstrated biocompatibility and biodegradability, silicon nanostructures represent promising candidates for electrical and optical neuromodulation as well [88]. The Tian group pioneered the use of silicon mesostructures for neuromodulation via the optocapacitive mechanism [85,89,90]. Compared to the AuNPs discussed above, silicon mesostructures are micron-sized particles that prevent receptor-mediated endocytosis. In addition, amorphous silicon benefits from enhanced light absorption, reduced rigidity and resistance to laser-induced particle change. Therefore, silicon mesostructures enable efficient and highly localized optocapacitive modulation of DRG neurons with remotely applied 532-nm laser pulses. In comparison with AuNPs, the authors demonstrated an enhanced photothermal efficacy in capacitive current generation by silicon mesostructures, as evidenced by an energy threshold around 30-fold less than that needed for previously reported AuNPs [85].

The optocapacitive mechanism requires a rapid temperature increase on the order of kilokelvins per second to drive a sufficient transmembrane capacitive current [91]. Such a steep slope of temperature increase precludes its application *in vivo* due to the limited ability to focus a pulsed laser through the scattering tissues. As an alternative to the non-specific optocapacitive mechanism, researchers have developed photothermal agents that target temperature-sensitive ion channels, such as TRPV1 and TRPA1 [92]. As a proof-of-concept *in-vitro* demonstration, the Pu group developed organic semiconducting polymer nanoparticles (SPNs) with strong absorption of NIR light (e.g. at 808 nm) [93]. In this work, SPNs were functionalized to bind TRPV1, thus potentially affording molecular specificity with less off-target effects. The authors demonstrated the advantages of SPNs over gold nanorods in terms of their better photothermal performance, characterized by the conversion efficiency, heating capability, stability and efficacy of cell modulation.

Another advantage of semiconducting polymers arises from their tunable bandgaps, which result in a broad range of absorption peaks in the first and second near-infrared windows (NIR-I and NIR-II windows, i.e. 700–900 nm and 1000–1700 nm, respectively) [12]. Taking advantage of biological tissue transparency in the NIR-II window, the Hong group recently reported *in-vivo* photothermal neuromodulation through the scalp and skull of freely behaving mice without any fiber implantations or head tethering (Fig. 7b) [19]. This advantage was achieved via macromolecular infrared nanotransducers for deep-brain stimulation (MINDS), conjugated polymer nanoparticles coated with an amphiphilic shell. Specifically, MINDS consisted of a semiconducting polymer core for absorbing NIR-II light at 1064 nm and a water-soluble and biocompatible polymer shell. As has been seen in Fig. 1e, 1064 nm is near the global maximum of the effective penetration depth of brain tissue, thus affording the deepest neuromodulation in the brain. The authors performed *in-vivo* studies to demonstrate the feasibility and specificity of NIR-II neuromodulation in three selected brain regions: the hippocampus, motor cortex and VTA. Specifically, an increase in the neural firing rate upon NIR-II illumination in the hippocampus via simultaneous electrophysiological recording was observed. Furthermore, behavior manipulation using MINDS and NIR-II light was also demonstrated in free-moving mice, exhibiting unilateral circling and CPP with photothermal neuromodulation in the secondary motor cortex and VTA, respectively. The sufficiency and necessity of TRPV1, MINDS and 1064-nm light were validated with electrophysiology, immunostaining and behavioral experiments. Notably, this demonstration represents the first time that photothermal neuromodulation has been applied in the deep brain of behaving animals in an implant-free and tether-free manner.

The energy conversion efficiency is a key parameter for all photothermal neuromodulation methods and is defined as the ratio of the generated thermal energy to the absorbed photon energy. Among an array of photothermal materials, the reported efficiency is distributed in a wide range from 19.2% to 98.9% [19,94,95]. However, the reader should be cautioned that a high photothermal efficiency does not imply a significant increase in temperature due to thermal diffusion to the environment where the photothermal agent is located. Specifically, to model the spatial distribution of temperatures with temporal dynamics, the Pennes bioheat equation should be solved numerically in live biological tissues [96]. In addition, since the photothermal efficiency only describes that of an internal process (akin to the ‘internal’ quantum efficiency of photovoltaics), the absorption cross section needs to be included when considering potential photothermal candidates. As a rule of thumb, materials that strongly absorb incident light and efficiently generate heat after absorption should be used for photothermal neuromodulation.

In addition to neuromodulation with the photothermal effect, the photoelectrochemical process, which generates Faradaic current under light illumination of certain materials, has also been leveraged for neuromodulation (Fig. 7c) [97]. For example, coaxial p-type/intrinsic/n-type (PIN) Si nanowires (SiNWs) with atomic Au on the surfaces were synthesized by the Tian group via a chemical vapor deposition process. Upon 532-nm light illumination, these hybrid nanostructures produced cathodic photocurrents sufficient to depolarize a targeted neuron. Specifically, electrons and holes migrated to the n-type shell and p-type core, respectively, thus driving Faradaic currents in a loop in response to light stimulation. Using these devices, the authors successfully induced action potentials in rat DRG neurons, which shared a similar waveform with those triggered by conventional current injection. To elucidate the mechanism of this method, the authors first ruled out the thermal effect as the main contributor by measuring a small temperature increase during laser illumination.

Taking a step further, the Tian group synthesized a series of silicon-based interfaces leveraging photoelectric (i.e. capacitive and Faradaic) and photothermal effects for multiscale interfacing with an array of biological systems including organelles, cultured neurons, brain slices and the live mouse brain [89]. On a subcellular level, SiNWs were found to not only serve as stimulators for modulating intracellular calcium, but also act as transport markers for labeling motor protein kinetics with 592-nm laser illumination. On a cellular level, extracellular photostimulation of 532-nm laser pulses successfully produced action potentials in cultured DRG neurons using a planar PIN junction in a Si diode. Additionally, on a tissue level, 473-nm photostimulation was used to produce excitatory postsynaptic currents (EPSCs) in *ex-vivo* brain slices via an array of PIN junctions in a mesh architecture. Finally, on the organism level, the authors successfully demonstrated 473-nm photostimulation of neural activity in the live mouse brain via an Au-coated PIN Si mesh array. This work represents a tour-de-force of non-genetic neuromodulation with Si nanostructures as multiscale optical neural interfaces based on a wide array of light-induced physical effects [89,98].

Beyond the mechanisms discussed above, photoacoustic stimulation represents an emerging technique for neuromodulation. Compared with conventional ultrasound neuromodulation methods [99], photoacoustic stimulation benefits from the much higher spatial resolution of light illumination (Fig. 7d) [100]. In one of the early demonstrations, optical fibers were used to deliver laser pulses, which led to efficient heat generation of light absorbing materials (e.g. graphite) and thermal expansion of the surrounding material (e.g. epoxy) with high spatial precision [101]. As a result, acoustic waves were generated to activate neurons, as evidenced by calcium imaging *in vitro* and electrophysiological recordings *in vivo*. A behavioral assay was also used to evaluate the evoked motor response and validate the high spatial resolution of this approach.

Besides high spatial resolution, photoacoustic neuromodulation also offers a unique tool for investigating the mechanism underlying ultrasound neurostimulation. Very recently, photoacoustic nanotransducers (PANs) comprising semiconducting polymer nanoparticles were developed for generating localized ultrasound upon NIR-II irradiation [100]. PANs were conjugated with antibodies to specifically target mechanosensitive TRPV4 channels, thus helping elucidate the mechanism of ultrasound modulation via activating these ion channels. In another recent work, a tapered fiber optoacoustic emitter (TFOE) was engineered for single-neuron photoacoustic stimulation [102]. The TFOE was fabricated by coating the fiber tip with carbon nanotubes (CNTs) in a PDMS matrix. Subcellular stimulation of neurites was demonstrated by 1030-nm laser pulses, which produced calcium wave propagation as observed in fluorescence imaging. Owing to the elimination of an ultrasound input, TFOE permitted simultaneous intracellular recording via the standard patch clamp technique, since light induces minimal mechanical disruption of the ‘gigaseal’ between the patch pipette and the cell membrane.

### Ultrasound- and magnetic-field-mediated light sources for optogenetics

All approaches discussed above rely either on light directly or on the secondary effects of light (e.g. photothermal, photoelectrochemical and photoacoustic). Nonetheless, a long-standing challenge of *in-vivo* light delivery remains unresolved; that is, due to the intrinsic scattering and absorption of light in biological tissues, invasive procedures are usually required to remove overlying tissues and implant optical fibers. Although red-shifted opsins (e.g. ChRmine) and NIR-responsive materials (e.g. UCNPs and MINDS) have demonstrated transcranial and even transdermal deep-brain stimulation with reduced invasiveness, transgene and nanoparticle delivery usually still requires invasive intracranial injection [19,21,22]. Addressing these challenges requires two biophotonic innovations: first, a nanotransducer is needed to convert tissue-penetrant energy, such as ultrasound and magnetic fields, into localized light emission; second, these nanotransducers are preferably delivered via intravenous instead of intracranial injection, since intravenous injection represents one of the simplest and least invasive methods for administering pharmacologics into the body.

**Figure 8.**
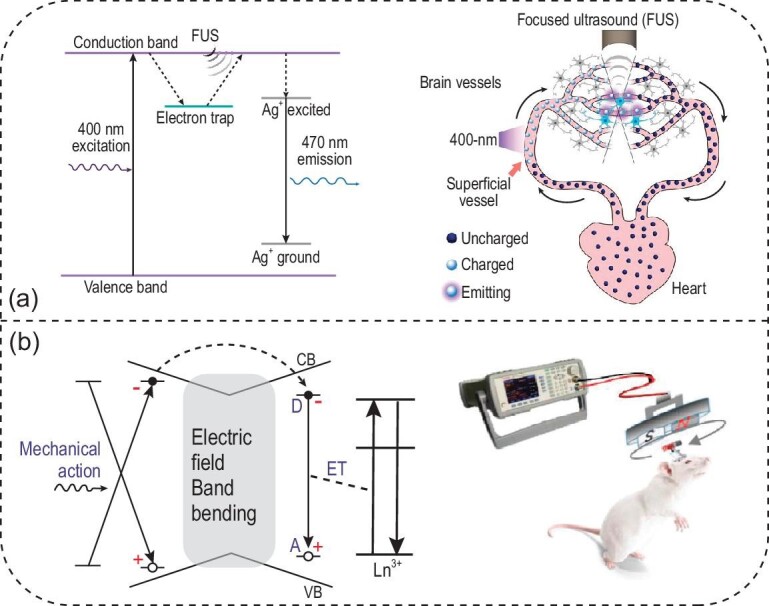
Ultrasound- and magnetic-field-mediated optogenetics. (a) The energy diagram of ZnS:Ag,Co enables efficient conversion of focused ultrasound (FUS) into 470-nm light emission (left). Furthermore, the endogenous circulatory system offers a minimally invasive means of delivering and recharging circulating ZnS:Ag,Co mechanoluminescent nanoparticles (right). Adapted with permission from [104]. Copyright 2020, American Association for the Advancement of Science. (b) The energy diagram of CaZnOS:Tb gives rise to mechanoluminescence under mechanical stress (left), which can be produced by a magnet bar under a rotating magnetic field for *in-vivo* optogenetics (right). Adapted with permission from [105]. Copyright 2020, Wiley‐VCH.

In the first attempt to produce localized emission with ultrasound, the Hong lab introduced the concept of ‘sono-optogenetics’ and demonstrated the proof-of-concept of this approach in live mice via a minimally invasive ultrasound interface [103]. Implementing this approach requires fulfilling the two biophotonic innovations above. On one hand, a nanotransducer is needed to convert tissue-penetrant ultrasound into localized light emission. To this end, the authors used defect engineering strategies to make zinc sulfide (ZnS) nanoparticles co-doped with trace amounts of silver (Ag) and cobalt (Co) ions (ZnS:Ag,Co) to afford a unique mechanoluminescence property. Upon 400-nm photoexcitation, Co dopant ions trap the excited electrons and store the photoexcitation energy until they are triggered by ultrasound. Ultrasound depletes the stored energy in the trap presumably via a piezoelectricity-induced mechanism and releases the energy as light emission. Finally, Ag dopant ions tune the emission color to 470-nm blue light to activate ChR2. In so doing, ZnS:Ag,Co mechanoluminescent nanoparticles can convert ultrasound, which has deeper tissue penetration (>1 cm for 1.5 MHz ultrasound vs. <1 mm for 470 nm light), to local light emission after photoexcitation, thus allowing us to metaphorically ‘see the sound’ (Fig. 8a, left) [104]. On the other hand, these nanotransducers can be delivered *in vivo* with minimal invasiveness by leveraging a unique endogenous mechanism offered by the circulatory system in the mammalian body. Specifically, after ZnS:Ag,Co NPs are administered intravenously, they can be recharged by photoexcitation in superficial vessels near the skin, pumped by the heart into deep-tissue vessels, and then gated by focused ultrasound to release the stored energy as visible light, all without exiting blood circulation (Fig. 8a, right). Using this circulation-delivered light source enabled by mechanoluminescent nanoparticles, the authors were able to evoke hindlimb motion synchronized with focused ultrasound (FUS) pulses in mice expressing ChR2 in the motor cortex.

Besides ultrasound, the Wang and Chang groups have demonstrated the production of light emission from mechanoluminescent materials by a rotating magnetic field [105]. A magneto-luminescence microdevice was developed, composed of terbium-doped calcium zinc oxysulfide (CaZnOS:Tb) crystals and a magnet bar (Fig. 8b). Under a rotating magnetic field, the magnet rotates in response and thus exerts mechanical stress on the CaZnOS:Tb crystals, producing light emission at 544 nm. This green emission arises from Tb^3+^ dopants and matches the activation spectrum of two optogenetic proteins, KillerRed and C1V1. Specifically, KillerRed is a genetically encoded protein that produces reactive oxygen species under green-light irradiation, thus inducing cancer cell death with remotely applied magnetic fields in both *in-vitro* and *in-vivo* conditions. Furthermore, C1V1 is a red-shifted channelrhodopsin that can be excited by the magneto-luminescence microdevice, enabling the authors to modulate neuron activity and animal behaviors in a wireless manner.

Since ultrasound- and magnetic-field-mediated light sources rely on energy stored in defects, such as electron and hole traps in the material, the density of these traps determines the theoretical limit on maximum energy that can be released by incident ultrasound or an external magnetic field. Although the exact mechanism of mechanoluminescence and the nature of energy-storing traps are still to be elucidated [106], the energy storage capacity of trap-engineered materials can be determined via time-dependent luminescence measurements in an integrating sphere. In one of the few studies of this kind, it was found that only ∼1.6% of the dopant, Eu^2+^ ions in a mechanoluminescent material, SrAl_2_O_4_:Eu,Dy, participates in energy storage [107]. Therefore, developing brighter light sources gated by ultrasound and magnetic fields depends on advancements that will produce more efficient optical storage materials. Taken together, both sono-optogenetics and magneto-luminescence microdevices demonstrate a new paradigm of *in-vivo* light delivery in deep tissues for minimally invasive optogenetics. We envision that the same light delivery approaches can be used in conjunction with other optical neuromodulation approaches and any biomedical applications that need a light source deep inside the body.

## SUMMARY AND OUTLOOK

Optical neuromodulation methods have transformed neuroscience and are continuing to inform new therapies for neurological and psychiatric diseases. Compared to the neural interfaces of other modalities, optical approaches benefit from diffraction-limited high spatial resolution, excellent temporal precision, the ability to propagate in free space, wavelength multiplexity, and orthogonality with electrical approaches. Owing to the unique ability of optogenetics to target distinct cell populations, spatiotemporally controlled illumination methods offer unique opportunities to dissect the neural circuits driving specific behaviors and disorders by turning neurons on and off with cell-type specificity.

An important consideration for choosing the appropriate optical methods for neuromodulation arises from the complex biological environments of various organs and structures in the central nervous system (CNS) and PNS. To guide the selection and further development of optical neuromodulation approaches, we summarize design considerations for suitable optical approaches in the context of technical challenges specific to each listed organ (Table 1). Additionally, we also point out some exciting new directions in optical neuromodulation methods in the following paragraphs.

**Table 1.** Challenges and opportunities for optical methods to modulate neural activity in different organs.

Nervous system	Organ	Reported optical approaches	Technical challenges	Design considerations
CNS	Brain	• Conventional optical fibers• Multifunctional fibers• Optical waveguides• Wired and wireless LED devices• Upconversion nanoparticles• Ultrasound- and magnetic-field-mediated light sources• Photothermal, photoelectrochemical and photoacoustic materials	• Dense neuronal density requires optical control with high spatial resolution.• Regions of interest are distributed in 3D, especially in the depth direction. The depth distribution of different brain targets imposes challenges to conventional illumination methods due to limited tissue penetration of visible light.• The interconnection between brain regions requires multisite optical control, ideally via a light source that can be repositioned *in operando*.• Neuroplasticity in the brain requires a chronically stable optical interface.	• Fine spatial control can be realized via taper optical fibers, spatially expandable fiber probes and reconfigurable nanophotonic silicon probes.• Ultrasound and NIR-II light offer deeper brain penetration for neuromodulation.• Focused ultrasound may facilitate raster scanning of the light emission spot inside the brain.• Flexible optoelectronics with reduced footprint enable a chronically stable optical interface for long-term neuromodulation.
	Spinal cord	• Conventional optical fibers• Multifunctional fibers• Wireless LED devices• Upconversion nanoparticles	• Mechanical motion is associated with natural movement of the subject.• The lack of the skull prevents a securely cemented interface.	• Flexible optoelectronics are required to conform to the curvilinear morphology of the spinal cord.• Stretchable optoelectronics accommodate mechanical motion.• Wireless interfaces are preferred due to the lack of an exposed surface for cementing a wired device.
PNS	Heart	• Conventional optical fibers• Wired and wireless LED devices	• Multiple neurons distributed over a large area need to be stimulated simultaneously for optogenetic pacing.• Cardiac motion may lead to an unstable optical interface.	• Stretchable film array with densely packed LEDs can conform to the surface of the myocardium.
	Bladder	• Conventional optical fibers• Wireless LED devices	• The dimensional change of the bladder due to its activity needs to be accommodated by the optical interface.	• Stretchable optoelectronics can accommodate the bladder volume change.
	Gut	• Wireless μLED devices• External light sources through an abdominal window	• Neurons are sparsely distributed through a large volume of abdominal tissue.• Constant bowel movements exist, thus placing challenges for a stable interface.• Different neuron types are segregated at different locations along the gastrointestinal tract, thus requiring region specificity of illumination.	• An internal light source is needed for a broad illumination area.• A relocatable light source is preferred to screen different neuron types at distinct locations in the gut.• A stretchable and conformal optoelectronic interface is desired to accommodate bowel movements.

We envision new methods for delivering light into the deep brain and the PNS with reduced invasiveness. Despite recent advances in transcranial optogenetics [19,21,22], it remains challenging to deliver light specifically to a deep-brain region while sparing all underlying brain tissue from irradiation. In addition, many challenges remain for optogenetics in the PNS. Specifically, the sparse distribution of neurons and nerve terminals over large areas, constant movement of the tissue, and the lack of cranial bone all represent technical challenges of optogenetic stimulation in the PNS [55,56]. Therefore, new methods for light delivery *in vivo*, especially those that allow for repositioning the illuminated region during experiments, will significantly benefit neuroscience studies in the PNS, such as in the gut [108,109]. Besides sono-optogenetics [103,104], which enables a circulation-delivered light source gated by external ultrasound, other internal light sources such as luciferase/luciferin offer another strategy of light delivery for optogenetics without an exogenous device [110]. Furthermore, ultrasound interference offers an elegant approach to modulating the local density of biological tissues, thus realizing a virtual GRIN lens for non-invasive light delivery. New knowledge about the complex interaction between tissue-penetrant energy (ultrasound [104], infrared light [19], X-ray [111] and radiofrequency [112,113]) and matter (endogenous tissues and exogenous materials) thus offers unique opportunities for next-generation *in-vivo* optical neuromodulation approaches.

We envision new methods will sensitize neurons to incident light, especially tissue-penetrant wavelengths in the NIR-I and NIR-II spectra, via endogenous and genetically encoded mechanisms. Although red-shifted opsin variants have been developed with sensitivity up to 635 nm, multi–vibrational-mode thermal statistics prohibit finding infrared-responsive opsins activated by longer wavelengths, at least in nature [114]. Nonetheless, inspired by the molecular basis of infrared detection by snakes, heat-sensitive transient receptor potential vanilloid (TRPV) ion channels, which can be genetically encoded in specific cell types, offer an alternative mechanism of infrared sensitivity [115]. This approach has already been demonstrated in transdermal NIR-II neuromodulation in the brain and engineered NIR vision in the retina [19,116]. In addition, to sensitize the neural tissue to photothermal stimulation, endogenous chromophores such as dopamine and melanin may be leveraged to produce efficient light absorbers in neurons with cell-type specificity, thus affording selective photothermal neuromodulation therein.

Besides these new possibilities, we also highlight the fundamental physical limitations when developing new materials and approaches for optical neural interfacing. The interaction between light and biological tissues, as discussed at the beginning of this review, dictates the limits in penetration depth and permissible power density for optical neuromodulation. Specifically, Fig. 1e reveals that a global maximum exists near 1070 nm for the effective attenuation length in the brain, which results from the compromise between decreasing scattering and increasing absorption at longer wavelengths. Therefore, the penetration depth of optical neuromodulation methods cannot be further optimized by going beyond the NIR-II window to reduce scattering according to Equation S12 (see Supplementary Data). This fundamental constraint limits the application of light-based approaches in larger-brained subjects such as humans without resorting to an invasive means. In addition, exogenous light-emitting devices cannot be miniaturized indefinitely due to size limitations: for example, as the size of μ-ILEDs shrinks, it becomes more challenging to epitaxially grow the light-emitting material on a smaller mesa, in addition to a higher density of defects that significantly impact the device performance [117]. As another example, mechanoluminescent nanoparticles emit light in response to ultrasound-induced mechanical stress, with the maximum light intensity theoretically limited by the number of trap energy states in the host materials [106]. Therefore, one must consider the physical principles governing neuromodulation when designing new optical neural interfaces with smaller footprints and higher throughput.

## Supplementary Material

nwac007_Supplemental_file
