# Supplementary material for: Shedding light on neurons: optical approaches for neuromodulation
Source: Natl Sci Rev. 2022 Jan 18;9(10):nwac007. doi: 10.1093/nsr/nwac007 (PMC9522429; doi:10.1093/nsr/nwac007)
Supplement: nwac007_Supplemental_file [file nwac007_supplemental_file.docx]

**Supplementary Information for**

**Shedding light on neurons: optical approaches for neuromodulation**

Shan Jiang^1,2^, Xiang Wu^1,2^, Nicholas J. Rommelfanger^2,3^, Zihao Ou^1,2^, and Guosong Hong^1,2^

^1^ Department of Materials Science and Engineering, Stanford University, Stanford, CA, 94305, USA

^2^ Wu Tsai Neurosciences Institute, Stanford University, Stanford, CA, 94305, USA

^3^ Department of Applied Physics, Stanford University, Stanford, CA, 94305, USA

^*^ Corresponding author: [guosongh@stanford.edu](mailto:guosongh@stanford.edu)

**1. Physical representations of light propagation.**

We use the following complex representation to describe the spatiotemporal variation of the electric field of a plane wave propagating in unbound media:

$\vec{E} =\vec{E_{0}}exp(i\vec{k}\cdot\vec{x}-i\omega t)$ (S1)

where $\vec{E}$ is the instantaneous electric field, $\vec{E_{0}}$ is the amplitude of the electric field in the propagating wave, $\vec{k}$ is the wave vector, $\vec{x}$ is the position vector, ω is the angular frequency, and *t* is time. We follow the convention of $e^{-i\omega t}$ for time-harmonic fields. Propagation of light in an absorbing medium, such as the brain, can be described mathematically by expressing $\vec{k}$ in the form of a complex vector

$\vec{k} =\vec{k'} +i\vec{k''}$ (S2)

where both $\vec{k'}$ and $\vec{k''}$ are real vectors. Here, $\vec{k'}$ contains the information of the phase velocity of light propagation, and as a result, the refractive index (RI) of the medium. In addition, $\vec{k''}$ reflects the absorption of light in the medium. The physical meanings of $\vec{k'}$ and $k''$ can be seen by replacing $\vec{k}$ with eq. (S2) in eq. (S1):

$\vec{\boldsymbol{E}} =\vec{E_{0}}exp(-\vec{k''}\cdot\vec{x})exp(i\vec{k'}\cdot\vec{x}-i\omega t)$ (S3)

Eq. (S3) clearly reveals that the absorption of light leads to the exponential decay of the electric field amplitude as light propagates in the medium, such as the brain. During light propagation, the magnetic field $\vec{H}$ is perpendicular to and in phase with $\vec{E}$. The magnitude of $\vec{H}$ oscillates in space and time in a similar manner to eq. (S3):

$\vec{\boldsymbol{H}} =\vec{H_{0}}exp(-\vec{k''}\cdot\vec{x})exp(i\vec{k'}\cdot\vec{x}-i\omega t)$ (S4)

The Poynting vector of this wave can be defined as follows:

$\vec{S}=\frac{1}{2}Re\{\vec{E}\times\vec{H^{*}}\}=\frac{1}{2\omega\mu}\left| \vec{E_{0}} \right|^{2}exp(-2\vec{k''}\cdot\vec{x})\vec{k'}$ (S5)

from which we see that the Poynting vector is in the same direction of $\vec{k'}$. Note that μ represents the permeability of the medium, which is assumed to be real in biological tissues. It is worth mentioning that the magnitude of the Poynting vector is called the irradiance or intensity of light at any given location or time. Dimensional analysis reveals that light irradiance (i.e., light intensity) should have a dimension of energy per unit area and time (i.e., J m^-2^ s^-1^ or W m^-2^ in SI units).

In most applications of optical neuromodulation, the propagation of a plane wave in a homogeneous medium in one dimension (1D) provides a sufficient theoretical framework. Therefore, eq. (S3) can be simplified in scalars:

$\vec{E}=\vec{E_{0}}exp(-k"z)exp(ik'z-i\omega t)$ (S6)

where we use *z* to denote the direction of 1D light propagation. Eq. (S6) also makes it clear that the phase velocity can be expressed as

$\boldsymbol{v=}\frac{\boldsymbol{\omega}}{\boldsymbol{k'}}$ (S7)

which thus fulfils the function of k’ to reflect the phase velocity and the real refractive index of the medium. The complex refractive index can be defined as

$\boldsymbol{N=n'+in"=}\frac{\boldsymbol{c}}{\boldsymbol{\omega/k}}\boldsymbol{=}\frac{\boldsymbol{ck}}{\boldsymbol{\omega}}\boldsymbol{=}\frac{\boldsymbol{c}}{\boldsymbol{\omega}}\boldsymbol{(k'+ik")}$ (S8)

Equating the real and imaginary parts of the complex refractive index with those of the complex wave vector yields

$\boldsymbol{n'=}\frac{\boldsymbol{c}}{\boldsymbol{\omega}}\boldsymbol{k'}$ (S9a)

$\boldsymbol{n"=}\frac{\boldsymbol{c}}{\boldsymbol{\omega}}\boldsymbol{k"}$ (S9b)

Using eqs. (S6) and (S9) to simplify eq. (S5) yields the expression of light intensity, which is defined as the magnitude of the Poynting vector:

$I=\frac{1}{2}Re\{\sqrt{\frac{\varepsilon}{\mu}}\}\left| \vec{E_{0}} \right|^{2}exp(-\frac{4\pi n"z}{\lambda})$ (S10)

in which λ is the wavelength of light.

**2. Scattering of light in the brain tissue**

Scattering represents a common behavior of light-tissue interaction in the brain. Scattering of light and any other electromagnetic waves in a medium is due to the heterogeneity of the refractive index *N* in the medium (recall eq. (S8)). For light-brain interactions, this heterogeneity usually occurs on the level of nanometers to microns as the result of the spatial separation of water and lipid molecules with distinct RI’s. For simplicity of discussion, we consider scattering of non-absorbing particles in a non-absorbing medium and thus use RI and the real component of *N* (i.e., *n*’) interchangeably. Specifically, a myelinated axon comprises an aqueous core with an RI of ca. 1.36 and an optically dense sheath with an RI of ca. 1.44.[^1^](https://paperpile.com/c/kBPilo/UFocF) In this example, the myelin sheaths act as scatterers in an aqueous medium, much akin to oil droplets in the aqueous phase of milk, thus effectively blocking the transmission of light through the brain.

The scattering (and absorption) of spherical particles in a medium can be solved mathematically by the Mie theory. A standard treatment of the Mie theory expands the electric and magnetic fields (as seen in eqs. (S1) and (S4), respectively) in vector spherical harmonics and applies boundary conditions between the sphere and the surrounding medium, which have different values of RI. Although the exact derivation is laborious, the most important and informative conclusions of the Mie theory lie in the expression of the scattering cross section, *C*_sca_. The scattering cross section is defined as the ratio of the power of scattering, *W*_sca_, to the incident light intensity, *I*, and can be solved as follows:

$C_{sca}=\frac{W_{sca}}{I}=\frac{2\pi}{k^{2}}\sum_{n=1}^{\infty} (2n+1)(\left| a_{n} \right|^{2}+\left| b_{n} \right|^{2})$ (S11)

where k is the scalar of the wave vector in the medium and is a real number due to our assumption of the non-absorbing medium. Furthermore, *a*_n_ and *b*_n_ are coefficients expressed in terms of spherical Bessel functions. When the size of the scatterer (e.g., the radius *a*) is much smaller than the wavelength of interest, i.e., a << λ, the scattering cross-section can be expressed as follows

$C_{sca}=\frac{128\pi^{5}N^{4}a^{6}}{3\lambda^{4}}\left| \frac{N_{p}^{2}-N^{2}}{N_{p}^{2}+2N^{2}} \right|^{2}$ (S12)

where N_p_ is the complex refractive index of the scattering particle. A 1/λ^4^ relationship can be seen for C_sca_, characteristic of the famous Rayleigh scattering for small particles. To a first approximation, eq. (S12) already suggests a strategy to reduce scattering in the brain: increasing the wavelength λ leads to a reduction of the scattering cross-section C_sca_. However, it is worth noting that many scatterers in the brain do not follow Rayleigh scattering (i.e., λ^-4^) due to their relatively large sizes in comparison to the wavelength, but they nonetheless exhibit a smaller C_sca_ at longer wavelengths.

The scattering cross section discussed above only deals with a single scattering particle in the medium. When considering the brain tissue as a macroscopic object, we need to take into account the density of scatterers (how many scatterers there are) in addition to the scattering cross section (how strongly each scatterer scatters light). Therefore, we can define the scattering coefficient μ_s_ as the product of the density of scatterers and C_sca_:

$\mu_{s}=\rho_{s}C_{sca}$ (S13)

where $\rho_{s}$ is the number of scatterers per unit volume. Similar to α, μ_s_ has the unit of (length)^-1^. We can thus interpret μ_s_ as the inverse of the scattering mean free path, i.e., the average distance between two consecutive scattering events. Therefore, light experiences more scattering events within the same path length in a more scattering medium with a large μ_s_. Due to fixed $\rho_{s}$ in a specific tissue, the wavelength dependence of μ_s_ reflects that of C_sca_, exhibiting a monotonic decrease with an increasing wavelength (**Fig. 1a**).[^2^](https://paperpile.com/c/kBPilo/PJInY)

The physical interpretation of μ_s_ in the context of the scattering mean free path provides a handy method for modeling scattering with a Monte Carlo simulation. Specifically, the Monte Carlo method simulates a random walk of photon packets through three-dimensional (3D) space.[^3^](https://paperpile.com/c/kBPilo/P69NX) For each photon packet traveling in the brain, it is assigned an initial energy to begin with, and its energy is updated based on the absorption of the medium and the distance it has traveled (discussed below). Specifically, the probability that a photon packet travels a free path of at least *s*_1_ without scattering is

$P(s\geq s_{1})=exp(-\mu_{s}s_{1})$ (S14)

Eq. (S14) can be differentiated with respect to s_1_ to yield the probability density function

$p(s_{1})=\frac{-dP(s\geq s_{1})}{ds_{1}}=\mu_{s}exp(-\mu_{s}s_{1})$ (S15)

The probability density function can then be sampled by a random number generator provided by a computer. Therefore,

$s_{1}=-ln(\xi)/\mu_{s}$ (S16)

where ξ is a random number in the range of (0,1). Eq. (S16) represents a useful relationship for sampling the step size of a photon packet in a scattering medium. Since each photon packet is assigned an energy, the step size obtained from eq. (S16) can be applied in eq. (S23) below to take into account the absorption of photon energy by the brain tissue (which equals the energy loss of the packet) during the free-path propagation between two scattering events.

Despite the facile linear approach for modeling scattering and absorption with the Monte Carlo simulation, there is a caveat for treating scattering and absorption as separate rather than interdependent processes. Importantly, it has been found that the scattering coefficient μ_s_ heavily depends on the absorption of the tissue, as evidenced by maxima in the scattering spectrum exactly matching those in the absorption spectrum.[^4^](https://paperpile.com/c/kBPilo/bXJGp) Although a plausible explanation attributes this interdependence to the anomalous dispersion near strong absorption bands, our preliminary results suggest an alternative mechanism due to the interaction between dipoles in the scatterers and optical transitions in the absorbing medium. Regardless of the exact mechanism, readers are cautioned that scattering coefficients used in Monte Carlo simulations are functions of absorption in the medium and should be updated based on the spatiotemporal heterogeneity of absorption in the brain tissue, which will be discussed below.

After each scattering event, the photon packet will travel in a different direction with a deflection angle θ between the original and the new directions. The scattering cross-section can also be expressed as a function of θ to indicate this anisotropy:

$C_{sca}(\theta)=\frac{8\pi^{5}N^{4}a^{8}}{\lambda^{4}r^{2}}\left| \frac{N_{p}^{2}-N^{2}}{N_{p}^{2}+2N^{2}} \right|^{2}(1+{cos}^{2}\theta)$ (S17)

Eq. (S17) shows that the measured effective scattering cross section of a single scatterer varies when a “virtual” detector moves to different polar angles θ and distances r with respect to the scatterer. One can verify that the integration of eq. (S17) over all solid angles in a sphere yields eq. (S12). Due to this anisotropy, photon packets tend to “remember” their original traveled direction after each scattering event (**Fig. 1a**, inset). The anisotropy factor *g* is introduced to quantify this “memory” effect of scattering, which can be measured quantitatively and equals the ensemble average of cosθ:[^5^](https://paperpile.com/c/kBPilo/a5Wn4)

$g=<cos\theta>$ (S18)

The probability distribution of cosθ is related to *g* as follows[^6^](https://paperpile.com/c/kBPilo/PpXcH):

$p(cos\theta)=\frac{1-g^{2}}{2{(1+g^{2}-2gcos\theta)}^{\frac{3}{2}}}$ (S19)

Using the same strategy to relate the probability density function with a random number ξ, one can sample cosθ as in

$cos\theta=\frac{1}{2g}\left[ 1+g^{2}-\left( \frac{1-g^{2}}{1-g+2g\xi} \right)^{2} \right]$ (S20)

where g is usually ~0.9 for brain tissue in the 400-1800 nm optical regime (**Fig. 1b**). The scattering coefficient μ_s_ and anisotropy factor g can be combined to yield a new quantity, the reduced scattering coefficient, μ_s_’:

$\mu_{s}^{'}=\mu_{s}\left( 1-g \right)$ (S21)

Therefore, μ_s_’ describes the diffusion of photon packets in a completely random walk with no “memory” of the previous direction of propagation after scattering. The inverse of μ_s_’ is the transport mean free path, the traveled distance of a photon packet after which the “memory” of the original propagation direction is eliminated (**Fig. 1c**, inset). Similar to μ_s_ , μ_s_’ also exhibits wavelength dependence: μ_s_’ decreases with an increasing λ (**Fig. 1c**).

**3. Absorption of light in the brain tissue**

Besides scattering, absorption represents another mechanism contributing to the attenuation of light in the brain tissue. The expression of light intensity in eq. (S10) clearly reflects the exponential decay of light intensity *I* in an absorbing medium, such as the brain tissue. One can define the absorption coefficient α as follows:

$\alpha=\frac{4\pi n"}{\lambda}$ (S22)

With this definition, eq. (S10) can be reduced to a more familiar form:

$I=I_{0}exp(-\alpha z)$ (S23)

where *I*_0_ combines all pre-exponential terms. The wavelength dependence of α of the brain tissue in the optical regime from 400 nm to 1800 nm is shown in **Fig. 1d**. As can be seen, the absorption spectrum of the brain has two prominent features: first, the absorption peaks below 600 nm are attributed to hemoglobins in the blood; second, several absorption peaks at 980 nm, 1200 nm, and 1450 nm correspond to the overtones of water molecules, which are abundant in all soft biological tissues.[^7^](https://paperpile.com/c/kBPilo/nBRxY) Apparently, specific brain regions may exhibit distinct absorption properties from the bulk brain due to the accumulation of certain chromophores. For example, neuromelanin gives rise to the black color of the substantia nigra (lit. “black substance” in Latin) in the brain, due to a broad absorption spectrum extending from the ultraviolet (UV) to the near-infrared (NIR) region.[^8^](https://paperpile.com/c/kBPilo/ZE0WZ) Besides neuromelanin, aromatic amino acid residues in proteins (e.g., tryptophan, tyrosine and phenylalanine), reduced nicotinamide adenine dinucleotide (NADH), and heterocyclic flavins slightly contribute to the overall optical absorption and its spatiotemporal heterogeneity in the brain as well.[^7^](https://paperpile.com/c/kBPilo/nBRxY) Therefore, it is necessary to consider the heterogeneity of light absorption in the brain tissue when designing optical neuromodulation methods targeting different brain regions.

A direct consequence of light absorption is the elevated temperature in the local brain region. Stujenske and Gordon et al. modeled light-induced heating in the brain tissue for optogenetics. Due to the strong absorption of 532-nm light in the brain (**Fig. 1d**), their model predicts a gradual temperature increase up to 2.2 ℃ within a few hundred microns of the light source at 10 mW output (e.g., an optical fiber).[^9^](https://paperpile.com/c/kBPilo/lPn3Z) Owen and Kreitzer et al. later found that this temperature increase alone can activate an inwardly rectifying potassium channel in the striatum, thus biasing behavioral results of optogenetic studies.[^10^](https://paperpile.com/c/kBPilo/fcNo3) Since a threshold intensity is usually required for opsin activation (e.g., 1 mW/mm^2^ for ChR2 and 10 mW/mm^2^ for NpHR)[^11,12^](https://paperpile.com/c/kBPilo/DO587+FBoeY) and since long periods of continuous illumination are commonly used for optogenetic inhibition, it is crucial to control for nonspecific heating due to tissue absorption in optical neuromodulation methods.[^13^](https://paperpile.com/c/kBPilo/dKBF7)

**4. Penetration depth of light in the brain tissue**

The discussion of scattering and absorption above lays the foundation for understanding the penetration depth of light in the brain tissue. For optical neuromodulation, the efficacy is determined by light intensity instead of coherence (from the perspective of wavefronts) and directionality (from the perspective of rays). Therefore, it is worth differentiating photons that have only undergone a few scattering events vs those that have traveled beyond the transport mean free path with completely smeared directions. Specifically, photons that only slightly deviate from the ballistic direction are called “snake” photons since they follow a “snake-like” path roughly in the original direction. These photons still penetrate in the depth direction, thus contributing to usable intensity for optical neuromodulation.[^14^](https://paperpile.com/c/kBPilo/aBJu6) A small portion of the snake photons are still ballistic in nature, thus carrying the information for forming a microscopic image of neural activity with diffraction-limited resolution. Since this review focuses on optical neuromodulation methods, the focus on ballistic photons is beyond our scope; however, it is worth noting that the connotations of “penetration depth” for optical neuromodulation and optical neural imaging are markedly dissimilar due to the differences between snake and ballistic photons. Fortunately, the penetration depth for optical neuromodulation is much greater than that for optical neural imaging, owing to the much larger population of snake photons than that of ballistic photons at elevated brain depths.

The effective penetration depth of incident light can be evaluated by applying energy conservation to the diffusion equation based on Fick’s Law. Similar to eq. (S12), when scattering is considered, light intensity decays exponentially as follows:

$I=I_{0}exp(-\frac{z}{\sqrt{\frac{D}{\mu_{a}}}})=I_{0}exp(-\frac{z}{\delta_{eff}})$ (S24)

where D is a proportionality factor called the diffusion constant and $\delta_{eff}$ is defined as the effective penetration depth. The Eddington approximation can be applied to obtain the expression of D when $\mu_{a}<<\mu_{s}(1-g^{2})$ in the following:

$D=\frac{1}{3\left( \mu_{a}+\mu_{s}' \right)}$ (S25)

Combining eq. (S24) and (S25) yields:

$\delta_{eff}=\frac{1}{\sqrt{3\mu_{a}(\mu_{a}+\mu_{s}')}}$ (S26)

Therefore, we can plot the effective penetration depth of light in the brain in the range of 400 to 1800 nm in **Fig. 1e**. Furthermore, the inverse of the effective penetration depth is usually referred to as the effective attenuation coefficient:

$\mu_{eff}=\frac{1}{\delta_{eff}}=\sqrt{3\mu_{a}(\mu_{a}+\mu_{s}')}$ (S27)

It is noteworthy that the maximum penetration depth is found at 1070 nm, which is close enough to the operation wavelength of the Nd:YAG laser, 1064 nm. The wide availability of Nd:YAG laser and the maximum penetration depth of light near its operation wavelength in the brain thus make this wavelength region (1050-1100 nm) attractive for deep-brain neuromodulation.[^15^](https://paperpile.com/c/kBPilo/Qzii8) As a comparison, we estimate the percentage of photons reaching a depth of 4 mm in the brain in **Fig. 1f**. Specifically, we choose four representative wavelengths relevant in optogenetics: 470 nm, which corresponds to the maximum activation of ChR2; 635 nm, which is the activation wavelength of opsins with the greatest red shift up to date[^16,17^](https://paperpile.com/c/kBPilo/kA4GP+2Kk5X); 980 nm, which is the wavelength used for deep-brain optogenetics with upconversion nanoparticles; and 1064 nm, which is a representative NIR wavelength at which the Nd:YAG laser operates. As expected, 1064-nm light attenuates by ~20 fold over a depth of 4 mm, in stark contrast to the attenuation of 10^9^ fold at 470 nm, 10^3^ fold at 635 nm, and 10^2^ fold at 980 nm over the same depth.

**5. Optical waveguides for light delivery**

The attenuation of light in the brain, which represents a combination of both absorption and scattering, prohibits efficient light delivery into the deep brain regions. To address this challenge, optical waveguides, such as optical fibers and dielectric slab waveguides are commonly used for illuminating a specific brain region with efficient light delivery. However, this illumination is only achieved at the cost of permanently damaging the brain tissue along the path of implantation.

Two requirements must be met to ensure efficient light delivery via an optical waveguide. First, there must be minimal attenuation of light during its propagation inside the waveguide. Second, the optical path of propagating light must be spatially confined within the optical waveguide (i.e., there must be minimal leakage of light from the sidewalls of the waveguide). In the following paragraphs, we discuss these two requirements from the perspective of light-matter interaction, similar to the discussion above for light-brain interaction.

To rationalize the first requirement (i.e., make waveguides with minimal light attenuation), the same arguments of absorption and scattering in preceding paragraphs can be used. Although a transparent material such as silica or plastic is used to draw the optical fibers that satisfy this requirement, “transparency” is usually discussed in the context of specific wavelengths; that is, materials that are transparent at certain wavelengths may absorb light strongly at others. The attenuation spectrum of low-loss optical fibers usually exhibits a decaying background from the visible to ca. 1400 nm region, which is attributed to Rayleigh scattering in inhomogeneous glass.[^18^](https://paperpile.com/c/kBPilo/lNXiF) This decaying background is followed by prominent absorption peaks in the infrared (1450 nm and beyond 1800 nm), which are attributed to the overtone bands of OH stretching modes.[^19^](https://paperpile.com/c/kBPilo/RiaAj) The same OH stretching overtone bands contribute to the NIR and IR absorption of water molecules, which are abundant in the neural tissue.[^7^](https://paperpile.com/c/kBPilo/nBRxY) The interplay between scattering and absorption leads to the “telecom” windows near 1550 nm, where light attenuation is minimal. Specifically, the transmission loss in the 1550-nm telecom window is ~0.2 dB/km, in contrast to that of >5.0 dB/km below 600 nm.[^18^](https://paperpile.com/c/kBPilo/lNXiF) For various fiber materials, potential absorption by chromophores and point defects (i.e., color centers), especially those produced photochemically in the fiber material in exposure to light (“fiber darkening”), should be taken into consideration when using them for biophotonic applications.[^20^](https://paperpile.com/c/kBPilo/bSW55)

To realize the second requirement, it is necessary to consider the behavior of electromagnetic waves at the boundary between two media with different RI’s. Applying Snell’s law allows us to find the critical angle, $\theta_{c}$, at and above which total internal reflection occurs:

$\theta_{c}=arcsin\frac{n_{2}}{n_{1}}$ (S28)

where n_1_ and n_2_ denotes the RI of the incident and refracted medium, respectively. Note that we drop the imaginary component of the RI due to the first requirement of minimal absorption in the waveguide. To spatially confine propagating light inside a waveguiding medium such as the optical fiber, a general requirement is

$n_{1}>n_{2}$ (S29)

where n_1_ and n_2_ represent the RI of the core and cladding material of the fiber, respectively. We will see how the inequality of (S29) is satisfied in a number of fiber optic devices for optogenetics later.

Besides optical fibers, another commonly used waveguide is the planar slab waveguide, which usually consists of a high RI dielectric layer surrounded on either side by low RI materials. Following the same argument as above, one can see that the high RI layer acts as the waveguiding medium with an RI of n_g_. Unlike the optical fiber in which the cladding layer is defined by a single RI, in the planar slab waveguide the waveguiding layer is usually surrounded by two layers of different RIs: we can define the RI of the substrate as n_s_, and that of the cover as n_c_. Solving the wave equation at the two interfaces for the transverse electric (TE) case yields the following solution at either interface:[^21^](https://paperpile.com/c/kBPilo/ou8c2)

$\vec{E}(x)=\vec{E_{0}}exp(\pm\sqrt{\beta^{2}-k^{2}n_{i}^{2}}x)$ (S30)

where $\beta$is the propagation coefficient along the longitudinal direction of the waveguide, and *k* is the scalar of the wave vector in the non-absorbing waveguide. Therefore, $\beta$ is also known as the longitudinal wavevector and simply the component of *k* in the longitudinal direction. Analysis of eq. (S30) reveals that guided wave must satisfy the following conditions to propagate within the slab waveguide:

$k{max(n}_{s},n_{c}) < \beta< kn_{g}$ (S31)

When the inequalities in (S31) are met, the propagating wave will be confined within the high RI layer (equivalent to the core of an optical fiber), and only evanescent waves decay exponentially in both the cover and substrate layers (equivalent to the cladding of an optical fiber).

**References**

(1) [Antonov, I. P.; Goroshkov, A. V.; Kalyunov, V. N.; Markhvida, I. V.; Rubanov, A. S.; Tanin, L. V. Measurement of the Radial Distribution of the Refractive Index of the Schwann’s Sheath and the Axon of a Myelinated Nerve Fiberin Vivo. *J. Appl. Spectrosc.* **1983**, *39* (1), 822–824.](http://paperpile.com/b/kBPilo/UFocF)

(2) [Yaroslavsky, A. N.; Schulze, P. C.; Yaroslavsky, I. V.; Schober, R.; Ulrich, F.; Schwarzmaier, H. J. Optical Properties of Selected Native and Coagulated Human Brain Tissues in Vitro in the Visible and near Infrared Spectral Range. *Phys. Med. Biol.* **2002**, *47* (12), 2059–2073.](http://paperpile.com/b/kBPilo/PJInY)

(3) [Wang, L.; Jacques, S. L.; Zheng, L. MCML—Monte Carlo Modeling of Light Transport in Multi-Layered Tissues. *Comput. Methods Programs Biomed.* **1995**, *47* (2), 131–146.](http://paperpile.com/b/kBPilo/P69NX)

(4) [Bashkatov, A. N.; Genina, E. A.; Kochubey, V. I.; Tuchin, V. V. Optical Properties of Human Skin, Subcutaneous and Mucous Tissues in the Wavelength Range from 400 to 2000 Nm. *J. Phys. D Appl. Phys.* **2005**, *38* (15), 2543.](http://paperpile.com/b/kBPilo/bXJGp)

(5) [Jacques, S. L.; Alter, C. A.; Prahl, S. A. Angular Dependence of HeNe Laser Light Scattering by Human Dermis. *Lasers Life Sci.* **1987**, *1* (4), 309–333.](http://paperpile.com/b/kBPilo/a5Wn4)

(6) [Henyey, L. G.; Greenstein, J. L. Diffuse Radiation in the Galaxy. *Astrophys. J.* **1941**.](http://paperpile.com/b/kBPilo/PpXcH)

(7) [Hong, G.; Antaris, A. L.; Dai, H. Near-Infrared Fluorophores for Biomedical Imaging. *Nature Biomedical Engineering* **2017**.](http://paperpile.com/b/kBPilo/nBRxY)

(8) [Grieco, C.; Kohl, F. R.; Hanes, A. T.; Kohler, B. Probing the Heterogeneous Structure of Eumelanin Using Ultrafast Vibrational Fingerprinting. *Nat. Commun.* **2020**, *11* (1), 4569.](http://paperpile.com/b/kBPilo/ZE0WZ)

(9) [Stujenske, J. M.; Spellman, T.; Gordon, J. A. Modeling the Spatiotemporal Dynamics of Light and Heat Propagation for In Vivo Optogenetics. *Cell Rep.* **2015**, *12* (3), 525–534.](http://paperpile.com/b/kBPilo/lPn3Z)

(10) [Owen, S. F.; Liu, M. H.; Kreitzer, A. C. Thermal Constraints on in Vivo Optogenetic Manipulations. *Nat. Neurosci.* **2019**, *22* (7), 1061–1065.](http://paperpile.com/b/kBPilo/fcNo3)

(11) [Klapoetke, N. C.; Murata, Y.; Kim, S. S.; Pulver, S. R.; Birdsey-Benson, A.; Cho, Y. K.; Morimoto, T. K.; Chuong, A. S.; Carpenter, E. J.; Tian, Z.; Wang, J.; Xie, Y.; Yan, Z.; Zhang, Y.; Chow, B. Y.; Surek, B.; Melkonian, M.; Jayaraman, V.; Constantine-Paton, M.; Wong, G. K.-S.; Boyden, E. S. Independent Optical Excitation of Distinct Neural Populations. *Nat. Methods* **2014**, *11* (3), 338–346.](http://paperpile.com/b/kBPilo/DO587)

(12) [Zhao, S.; Cunha, C.; Zhang, F.; Liu, Q.; Gloss, B.; Deisseroth, K.; Augustine, G. J.; Feng, G. Improved Expression of Halorhodopsin for Light-Induced Silencing of Neuronal Activity. *Brain Cell Biol.* **2008**, *36* (1-4), 141–154.](http://paperpile.com/b/kBPilo/FBoeY)

(13) [Cardozo Pinto, D. F.; Lammel, S. Hot Topic in Optogenetics: New Implications of in Vivo Tissue Heating. *Nat. Neurosci.* **2019**, *22* (7), 1039–1041.](http://paperpile.com/b/kBPilo/dKBF7)

(14) [Dunsby, C.; French, P. M. W. Techniques for Depth-Resolved Imaging through Turbid Media Including Coherence-Gated Imaging. *J. Phys. D Appl. Phys.* **2003**.](http://paperpile.com/b/kBPilo/aBJu6)

(15) [Wu, X.; Jiang, Y.; Rommelfanger, N. J.; Yin, R.; Liu, J.; Cai, S. Through-Scalp Deep-Brain Stimulation in Tether-Free, Naturally-Behaving Mice with Widefield NIR-II Illumination. *bioRxiv* **2020**.](http://paperpile.com/b/kBPilo/Qzii8)

(16) [Marshel, J. H.; Kim, Y. S.; Machado, T. A.; Quirin, S. Cortical Layer–specific Critical Dynamics Triggering Perception. **2019**.](http://paperpile.com/b/kBPilo/kA4GP)

(17) [Chen, R.; Gore, F.; Nguyen, Q.-A.; Ramakrishnan, C.; Patel, S.; Kim, S. H.; Raffiee, M.; Kim, Y. S.; Hsueh, B.; Krook-Magnusson, E.; Soltesz, I.; Deisseroth, K. Deep Brain Optogenetics without Intracranial Surgery. *Nat. Biotechnol.* **2020**. https://doi.org/](http://paperpile.com/b/kBPilo/2Kk5X)[10.1038/s41587-020-0679-9.](http://dx.doi.org/10.1038/s41587-020-0679-9.)

(18) [Chen, J.-H.; Xiong, Y.-F.; Xu, F.; Lu, Y.-Q. Silica Optical Fiber Integrated with Two-Dimensional Materials: Towards Opto-Electro-Mechanical Technology. *Light Sci Appl* **2021**, *10* (1), 78.](http://paperpile.com/b/kBPilo/lNXiF)

(19) [Lange, K. R.; Wells, N. P.; Plegge, K. S.; Phillips, J. A. Integrated Intensities of O−H Stretching Bands: Fundamentals and Overtones in Vapor-Phase Alcohols and Acids. *The Journal of Physical Chemistry A*. 2001, pp 3481–3486. https://doi.org/](http://paperpile.com/b/kBPilo/RiaAj)[10.1021/jp003277u.](http://dx.doi.org/10.1021/jp003277u.)

(20) [Dragic, P. D.; Cavillon, M.; Ballato, J. Materials for Optical Fiber Lasers: A Review. *Applied Physics Reviews* **2018**, *5* (4), 041301.](http://paperpile.com/b/kBPilo/bSW55)

(21) [Pollock, C. R.; Lipson, M. *Integrated Photonics*; Springer, Boston, MA, 2003.](http://paperpile.com/b/kBPilo/ou8c2)
